# Time varying causal network reconstruction of a mouse cell cycle

**DOI:** 10.1186/s12859-019-2895-1

**Published:** 2019-05-29

**Authors:** Maryam Masnadi-Shirazi, Mano R. Maurya, Gerald Pao, Eugene Ke, Inder M. Verma, Shankar Subramaniam

**Affiliations:** 1 Department of Electrical and Computer Engineering and Bioengineering, University of California San Diego, 9500 Gilman Dr, La Jolla, CA 92093 USA; 2 Department of Bioengineering and San Diego Supercomputer center, University of California San Diego, 9500 Gilman Dr, La Jolla, CA 92093 USA; 3 Salk institute for Biological Studies, 10010 N Torrey Pines Rd, La Jolla, CA 92037 USA; 4 Department of Bioengineering, Departments of Computer Science and Engineering, Cellular and Molecular Medicine, and the Graduate Program in Bioinformatics, University of California San Diego, 9500 Gilman Dr, La Jolla, CA 92093 USA

**Keywords:** Dynamics, Cell cycle, Time series, Change point detection, Time varying network reconstruction, Causal inference, Temporal variation

## Abstract

**Background:**

Biochemical networks are often described through static or time-averaged measurements of the component macromolecules. Temporal variation in these components plays an important role in both describing the dynamical nature of the network as well as providing insights into causal mechanisms. Few methods exist, specifically for systems with many variables, for analyzing time series data to identify distinct temporal regimes and the corresponding time-varying causal networks and mechanisms.

**Results:**

In this study, we use well-constructed temporal transcriptional measurements in a mammalian cell during a cell cycle, to identify dynamical networks and mechanisms describing the cell cycle. The methods we have used and developed in part deal with Granger causality, Vector Autoregression, Estimation Stability with Cross Validation and a nonparametric change point detection algorithm that enable estimating temporally evolving directed networks that provide a comprehensive picture of the crosstalk among different molecular components. We applied our approach to RNA-seq time-course data spanning nearly two cell cycles from Mouse Embryonic Fibroblast (MEF) primary cells. The change-point detection algorithm is able to extract precise information on the duration and timing of cell cycle phases. Using Least Absolute Shrinkage and Selection Operator (LASSO) and Estimation Stability with Cross Validation (ES-CV), we were able to, without any prior biological knowledge, extract information on the phase-specific causal interaction of cell cycle genes, as well as temporal interdependencies of biological mechanisms through a complete cell cycle.

**Conclusions:**

The temporal dependence of cellular components we provide in our model goes beyond what is known in the literature. Furthermore, our inference of dynamic interplay of multiple intracellular mechanisms and their temporal dependence on one another can be used to predict time-varying cellular responses, and provide insight on the design of precise experiments for modulating the regulation of the cell cycle.

**Electronic supplementary material:**

The online version of this article (10.1186/s12859-019-2895-1) contains supplementary material, which is available to authorized users.

## Background

The progression of a eukaryotic cell cycle is governed by a complex, dynamical network of molecular interactions that regulate a series of directional and irreversible events such as cell growth, DNA replication, mitosis, and cell division. The biochemical pathways controlling the order and timing of cell cycle phases play an essential role in maintaining genomic stability of the cell. Significant progress has been made in identifying molecular players and pathways involved in cell cycle mechanisms through extensive investigations on model systems such as yeast. Protein assays, transcriptional studies, fluorescent imaging, and protein interaction mapping have all contributed to our current understanding of the cell cycle. From these studies and other phenotypic assays, molecular players engaged in distinct phases of the cell cycle, namely, G1, S, G2, and M phases, have been identified, resulting in a static pathway map of the cell cycle [1]. These maps lack dynamical information, owing to the absence of systematic time series measurements. *In-silico* experiments have helped researchers develop mathematical models that characterize the dynamics of cell cycle in yeast and other eukaryotic cells [2–4]. In addition, fine-grained time series measurements of a mammalian cell cycle can enrich the understanding of dynamical networks through which the temporal relationships between molecular players can be inferred, and further provide insights into mechanistic causality. In this work, we present a systematic fine-grained RNA sequencing study of the transcriptional profiles during a mammalian cell cycle.

Inferring causality from time-series data poses considerable challenges; conventional methods of network reconstruction offer a static characterization of the network topologies. For example, correlation-based methods [5, 6], matrix-based methods such as least-squares, principal component regression (PCR) [7], and partial least squares (PLS) [8], L1-penalty based approaches such as least absolute shrinkage and selection operator (LASSO) and fused LASSO [9, 10], Gaussian graphical models [11], and information-theory based approaches [12, 13] are among the methods primarily used for static network reconstruction. Boolean network (BN) is used to model dynamic gene regulatory networks through parameter estimation [14–16], however it requires discretization of gene expression levels to binary values to permit parameter estimation. Dynamic Bayesian learning approach provides a temporally evolving picture of the network [17, 18], but is computationally expensive and tends to perform poorly on high dimensional data. Even though time series data can be used to easily construct correlation networks, developing quantitative models from these data is complicated due to the inherent nonlinearity of biological systems. However, it is possible to capture this nonlinearity using successive linear models over distinct time windows or temporal regimes. The assumption is that within a given regime, the topology of the network does not change. While there has been several attempts at identifying different regimes in long time-series, mainly in the signal processing community [19–21], they have not been used to further develop evolving dynamical models and networks for biological systems.

We have developed a framework to investigate the temporal changes in the cell cycle network using RNA-seq time series data from Mouse Embryonic Fibroblast (MEF) primary cells. We use a non-parametric change point detection (CPD) algorithm [22] based on Singular Spectrum Analysis (SSA) [23] to infer the mechanistic changes in the time-course data for a set of 63 cell cycle genes to estimate cell cycle phases. We also use the notion of Granger causality implemented through a vector autoregressive (VAR) model [24] to predict the future expression levels of each gene as a function of the past expression levels of other genes yielding directionality of gene regulation among the 63 cell cycle genes. Furthermore, we utilize the concept of Minimum Description Length (MDL) to use past expression levels of genes, up to 9 time lags (equivalent to 4.5 h), to determine the minimum data information from past events required for a robust prediction of values at the current time.

This computational scheme enabled us to (i) estimate the timing of cell cycle phases, (ii) infer the duration of the G1, S and G2/M phases of the MEF cell cycle to be 14.5, 10 and 4 h, respectively, (iii) reconstruct three successive directed graphs representing the key regulatory mechanisms among the 63 cell cycle genes in the G1, S and G2/M phases of the cell cycle, (iv) infer the temporal impact that biological processes have on one another, as well as the dynamic changes in temporal dependencies as the cell evolves through successive phases, and (v) reflect the chronological order of regulatory events that are crucial to cell cycle control. The main power of our work is its ability to capture important causal interactions over time, providing a broad picture of the dynamics of a cell cycle regulatory network. We validate the reliability of our time-varying network for cell cycle progression by comparing the interactions detected in our results to the well-known regulatory pathways in the literature.

## Results

Gene expression in MEFs is measured at 96 different time points at intervals of 0.5 h or 1 h (later interpolated to every 0.5 h), covering more than one full cycle and the G1, S and part of G2/M phases of another cycle. Of the 4248 differentially expressed genes, i.e., genes whose expression values change more than 2-fold as compared to that at t = 0 at one or more time points, 63 are cell-cycle genes included in the Kyoto Encyclopedia of Genes and Genomes (KEGG) pathways database [1]. We first detected the different stages of the cell cycle using the CPD algorithm. Then we developed a VAR model for each stage through the estimation of optimal time-lags. Finally, we carried out an in-depth analysis of the temporally evolving networks as the cell cycle progresses.

### Detecting temporal changes and stages in the cell cycle time series data

**Fig. 1:**
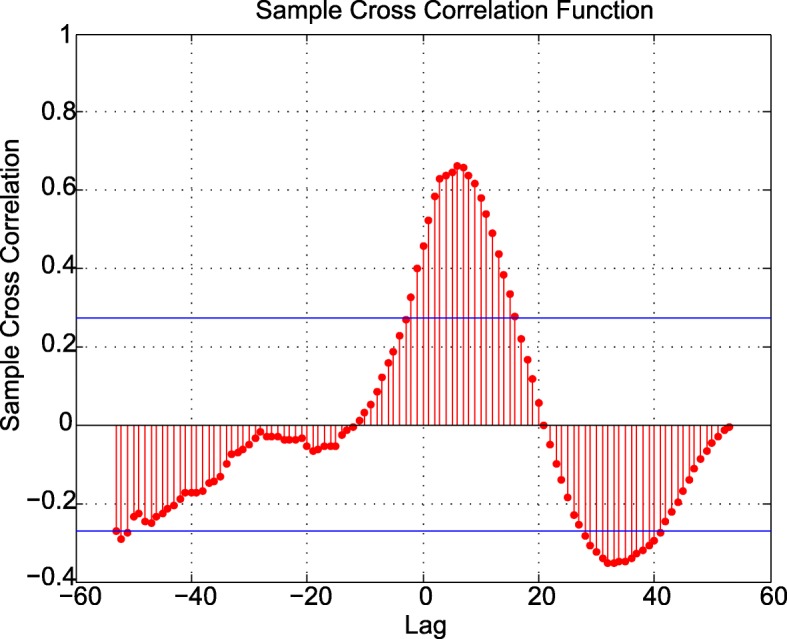
Cross correlation of two time-series of Smc1a gene. The cross correlation plot of the two time-series shows that maximal association for the two time-series occurs with an offset of 7 samples

In order to identify different phases of the cell cycle from the time-series data, we use a model-free CPD algorithm (discussed in the Methods section) [22]. The CPD algorithm captures the ongoing mechanistic changes as the cell cycle progresses and partitions the time series data into intervals with dominant trends, associated with cell cycle phases. It can be noted that no a priori assumptions on the duration of the cell cycle phases were incorporated in our analysis. In this study, we apply the CPD algorithm to 63 cell cycle genes presented in the KEGG pathway for mouse cell cycle [1] (Additional file 1 presents the list of genes). For every gene, the time-course data for approximately two consecutive cell cycles are available. We use cross-correlation between the two time-series data to obtain the offset between the two cycles by finding the time point at which the maximum association between the two time-series occurs (see Fig. 1 and supplementary methods in Additional file 2).

**Fig. 2:**
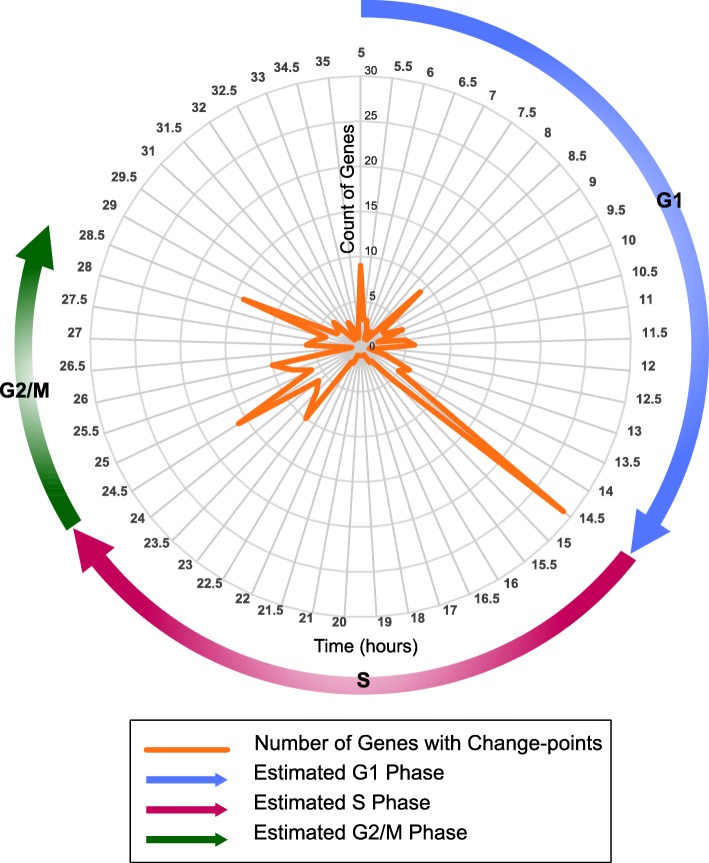
Segmentation of MEF cell cycle data with the change-point detection algorithm. Radar chart displays the count of genes that were detected to have change points at every sample (1/2 *h*) in the gene expression profiles of the 63 cell cycle genes

When the offset is computed for every gene, the gene expression profile is derived by properly concatenating the two time-series according to the offset and then the CPD algorithm is applied. This algorithm may detect more than one change point in the expression profile of each of the 63 cell cycle genes. Figure 2 is a radar chart that depicts the count of genes for which the CPD algorithm detects change points at every time point (1/2 *h*) (data from 5 h to 35 h after the start of the first cell cycle is shown in Fig. 2). There are three significant peaks in the radar chart at 14.5, 24.5 and 28.5 h at which the CPD algorithm detects change points for 29, 16 and 14 genes, respectively. We consider these peaks as break-points between the consecutive G1, S and G2/M phases of the cell cycle. According to the radar chart in Fig. 2, the duration of the G1, S and G2/M phases of the cell cycle is estimated to be 14.5, 10 and 4 h, respectively. Therefore, we associate the intervals {1–14.5}, {14.5–24.5} and {24.5–28.5} *hours* to the expression profile of genes in the G1, S and G2/M phases of the cell cycle.

### Network reconstruction from cell cycle time-series data

After detection of the major temporal intervals associated with cell cycle phases, the successive directed graphs reflecting causal relationships of 63 cell cycle genes are reconstructed as the cell progresses through the G1, S and G2/M phases. In this work, the notion of Granger causality is used to predict directionality of links in the networks. Based on the definition of Granger causality, a series *X*(*t*) is said to cause series *Y*(*t*) if the future value of *Y*(*t*) is better predicted using the past values of *X*(*t*) and *Y*(*t*) than when the future value of *Y*(*t*) is predicted using only the past values of itself [25]. With the assumption that gene expressions may be modeled through a linear regression, one can identify Granger causality through Vector Autoregressive (VAR) models (see Methods section). A *d*-order VAR model of a *k* dimensional time series is given by:

1 $$ y(t)=v+{A}_1y\left(t-1\right)+{A}_2y\left(t-2\right)+\dots +{A}_dy\left(t-d\right)+{\varepsilon}_t;t=0,1,\dots, T\kern1.75em $$

where *y*(*t*) is a vector of realization of random variables at time *t*, and *y*(*t* − *d*) at *d* samples before time *t*. Since the VAR model can be of any arbitrary order 1, 2, … *d*, the question of what the optimal order is arises. The optimal order of a variable *y*_*i*_(*t*) in the VAR model determines the number of time-lags that is necessary to take into account, in order to extract sufficient information from the lagged values of all variables that can provide the most accurate prediction of *y*_*i*_(*t*). This optimal order is estimated with the Minimum Description Length (MDL) principle [26]. Description length (DL) is a measure of trade-off between the residual sum of squares (RSS) and the complexity (order) of the VAR model:

2 $$ DL=\frac{T}{2}\log RSS+\frac{d}{2}\log T;d=1,2,\dots, {d}_{max} $$

**Fig. 3:**
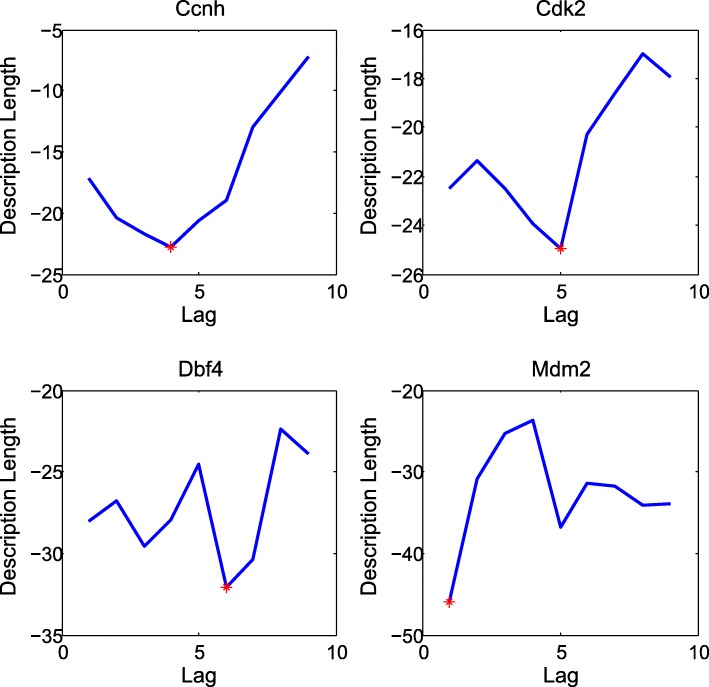
The plot of the description length for up to order *d*_*max*_ = 9 in the estimated G1 phase. The optimal order, shown in a red asterisk, is the order at which the description length is minimized. As shown, the description length is minimized when the expression profiles of Ccnh, Cdk2, Dbf4 and Mdm2 are modeled through VAR models of order 4, 5, 6 and 1, respectively

The MDL selects the optimal order such that the description length is minimized. Here we compute the description length of the VAR model for each gene separately up to order *d*_*max*_ = 9. Figure 3 shows the plot of the description length of four genes in the estimated G1 phase.

**Fig. 4:**
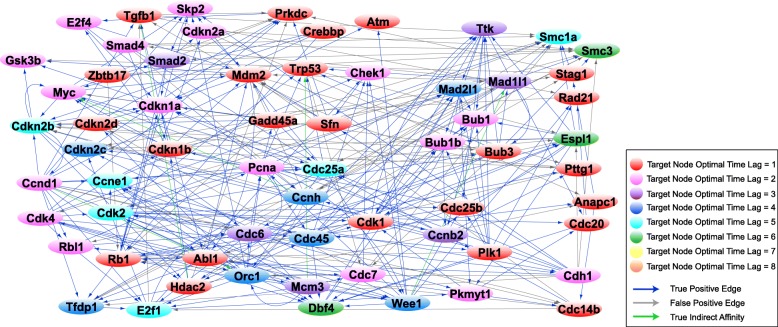
MEF cell cycle network for G1 phase. The graph reconstruction of the network representing the causal interactions of 63 cell cycle genes obtained by using only the data samples in the interval {1–14.5} *hour* of the cell cycle associated with the G1 phase. The blue edges represent true positive (TP) connections validated though the known literature (STRING database). The green edges represent true indirect affinities between the pairs of genes they are connected to, and the gray edges are interactions captured only in our model, serving as potential novel hypotheses. The node colors denote the optimal time lag corresponding to every target gene in the VAR model. See Additional file 3 for the complete list of interactions in the above network

**Fig. 5:**
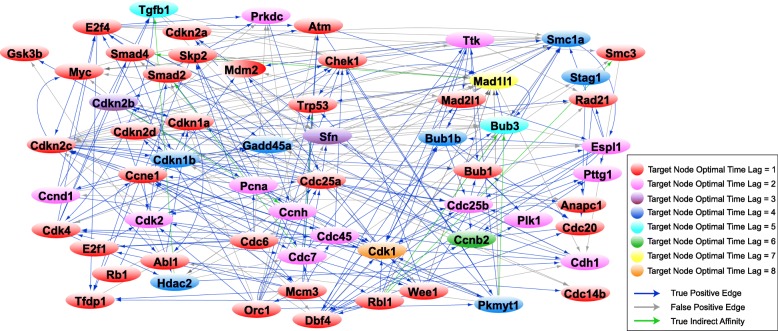
MEF cell cycle network for G1 phase followed by the S phase. The graph reconstruction of the network representing the causal interactions of 63 cell cycle genes obtained by using only the data samples in the interval {1–24.5} *hour* of the cell cycle associated with the S phase. The blue edges represent true positive (TP) connections validated though the known literature (STRING database). The green edges represent true indirect affinities between the pairs of genes they are connected to, and the gray edges are interactions only in our model, serving as potential novel hypotheses. The node colors denote the optimal time lag corresponding to every target gene in the VAR model. See Additional file 4 for the complete list of interactions in the above network

**Fig. 6:**
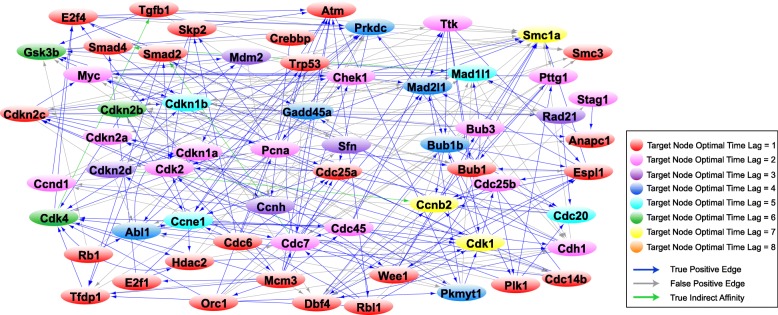
MEF cell cycle network for G1 and S phases followed by the G2/M phases. The graph reconstruction of the network representing the causal interactions of 63 cell cycle genes obtained by using only the data samples in the interval {1–28.5} *hour* of the cell cycle associated with the G2/M phase. The blue edges represent true positive (TP) connections validated though the known literature (STRING database). The green edges represent true indirect affinities between the pairs of genes they are connected to, and the gray edges are interactions captured only in our model, serving as potential novel hypotheses. The node colors denote the optimal time lag corresponding to every target gene in the VAR model. See Additional file 5 for the complete list of interactions in the above network

Once the optimal order for each gene is computed through MDL, we reconstruct three successive networks that reveal the evolution of the gene regulatory network of the 63 cell cycle genes through a complete cell cycle. Towards this, we use the expression profiles of genes for the three intervals {1–14.5}, {1–24.5}, and {1–28.5} *hours* derived through the CPD algorithm. Figure 4 depicts the gene regulatory network related to the {1–14.5} *hour* interval of the cell cycle associated with the G1 phase, Fig. 5 shows the network reconstructed for the {1–24.5} *hour* interval associated with the G1 phase followed by the S phase, and Fig. 6 illustrates the network representing the {1–28.5} *hour* interval related to the complete cell cycle (G1 and S phases followed by the G2/M phase). The resulting interactions have been validated with prior literature and the interactions in the STRING database. Table 1 presents the precision and false discovery rate of predictions in the reconstructed networks in Figs. 4, 5 and 6.

**Table 1:** Statistics for the reconstructed network of the G1, S and G2 phases in Figs. 4, 5, and 6

Reconstructed network	Number of true positive edges	Number of false positive edges	Precision	False Discovery Rate
G1 phase	268	76	0.78	0.22
S phase	198	78	0.72	0.28
G2/M phase	203	103	0.61	0.39

### Temporal dependence of biological processes in the cell cycle

**Fig. 7:**
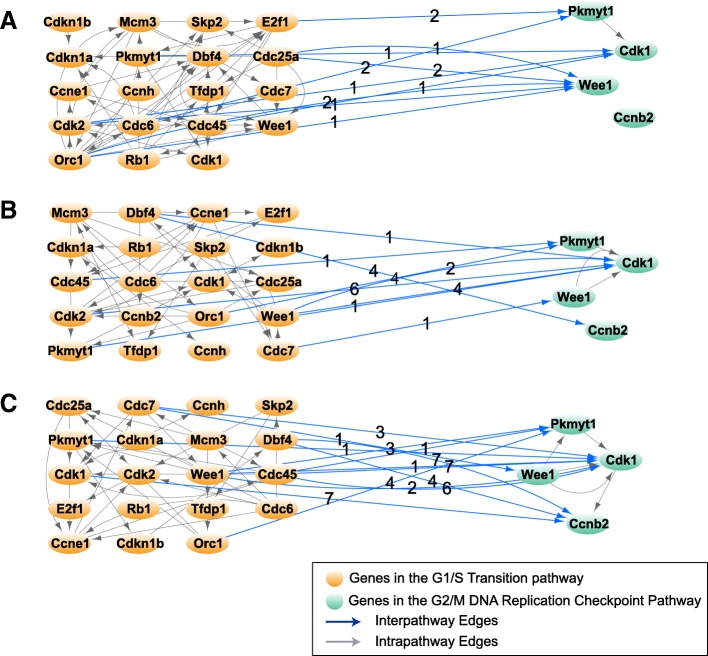
Temporal dependence of G2/M DNA replication checkpoint mechanism on the G1/S transition mechanism. Orange nodes are genes that take part in G1/S transition mechanism of the cell cycle and the green nodes are genes that take part in G2/M DNA replication pathway. Every edge label denotes the temporal dependence of the target node on the source node. In this example, the farthest dependence is 7 time lags. **a** Temporal dependence of G2/M DNA replication pathway on the G1/S-transition pathway in the {1–14.5} *hour* interval. **b** Temporal dependence of G2/M DNA replication pathway on the G1/S-transition pathway in the {1–24.5} *hour* interval. **c** Temporal dependence of G2/M DNA replication pathway on the G1/S-transition pathway in the {1–28.5} *hour* interval

In order to understand the temporal aspect of cell cycle processes, we analyze the transient length of influence of dynamic processes on one another; our primary question seeks to ask if one biological event induces the occurrence of another event in the cell, what is the duration of its influence? We sought to explore the temporal dependence of intracellular processes by considering 16 time-dependent biological processes governing the progression of the cell cycle. Additional file 2: Table S1 shows these biological mechanisms listed in the chronological order of their occurrence during a cell cycle along with their members (genes) according to the Reactome pathway database [27]. In the three successive networks in Figs. 4, 5, and 6, we group cell cycle genes that belong to each of the 16 biological processes into modules and infer the temporal dependence of modules on one another. The temporal dependence of these processes are assessed by taking into account the average of directed edge time-lags between pairs of processes. For instance, Fig. 7a, b, and c display the links from the nodes in G1/S transition module to the nodes in the G2/M DNA replication checkpoint mechanism as the cell goes through the G1, S, and G2/M phases, respectively. The numbers labeling these links denote the optimal number of time-lags required in the VAR model when assessing Granger causality.

**Fig. 8:**
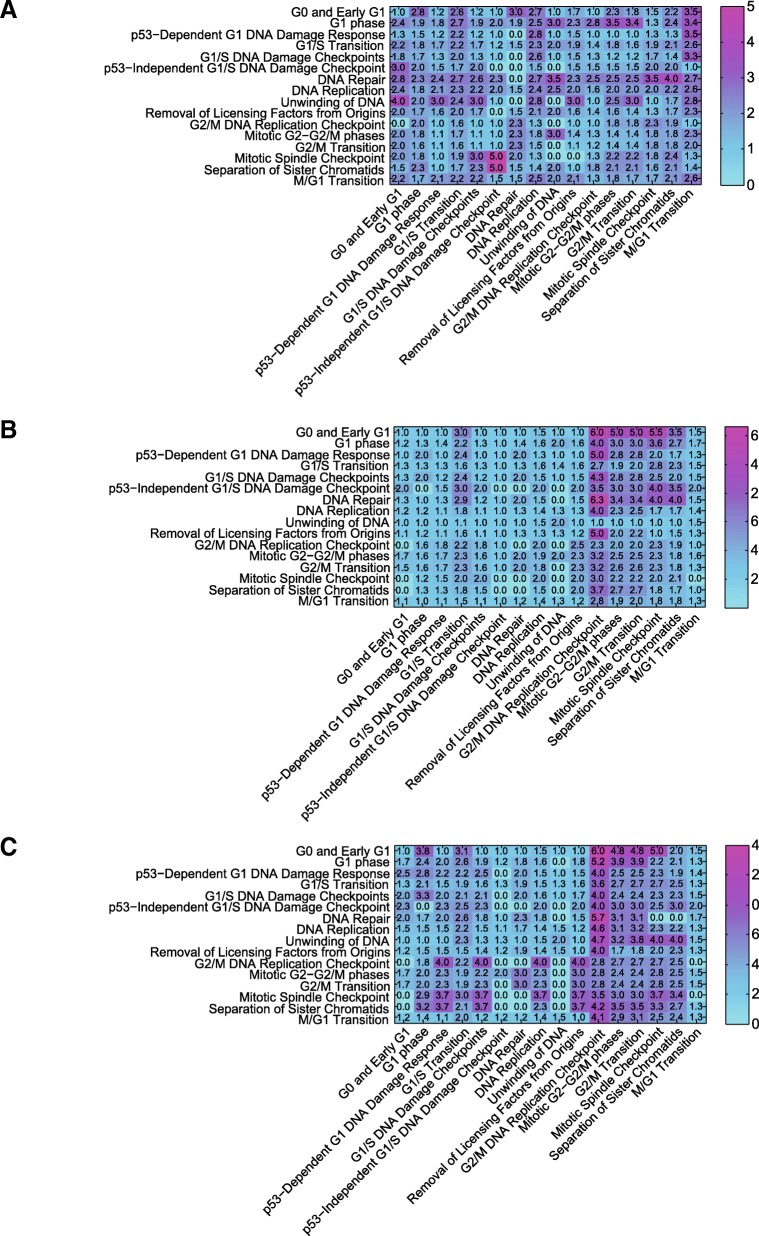
Temporal interdependencies of biological processes as the cell goes through the G1, S and G2/M phases. Each row and column in the heat map represents one of the 16 time-dependent biological processes. The number in every pixel represents the average time-lag of edges sourcing from its corresponding row process and targeting its column process (one lag is equivalent to ½ hour). **a** Heatmap of temporal dependence of processes as the cell goes through the G1 phase, **b** Heatmap of temporal dependence of processes as the cell goes through the G1 followed by the S phase. **c** Heatmap of temporal dependence of processes as the cell goes through the G1, S and G2/M phases

The average time-lags of edges in the three graphs in Fig. 7a, b, and c are 1.4, 2.67, and 3.62, respectively. As the cell evolves through a complete cell cycle, the average time-lag of the causal effect the G1/S transition mechanism has on the G2/M DNA replication mechanism increases. To further explore the length of intertwined temporal dependence these biological processes have on one another, we extend this analysis to all 16 intracellular processes listed in Additional file 2: Table S1. Fig. 8a, b, and c show the heat map plots displaying the average time-lag of edges between each pair of the 16 processes as the cell completes the G1, S, and G2/M phases. The heat map images identify temporal dependence of biological events on one another in different stages of the cell cycle.

### G1 phase

The G1 phase, also known as the Gap 1 phase, is the first of the four phases that occur in one complete eukaryotic cell cycle. During the G1 phase, the cell grows in size and synthesizes mRNA and proteins required for DNA synthesis. In this section, we investigate the role of key regulatory proteins and their corresponding phase specific interactions found in the reconstructed G1 phase network (Fig. 4). The complete list of the edges estimated in the G1 phase network is presented in Additional file 3.

#### Rb1/Rbl1

In Fig. 4, we note Rb1 interacts with Cdkn1a, Cdkn2a, Skp2, Cdh1, and Anapc1. It is known that Cdkn1a forms a physical complex with Rb1 and can activate Rb1 to bring about cell cycle arrest [28, 29]. Furthermore, Rb1 activity is mainly regulated by Cdkn2a inhibition of Ccnd1 to prevent phosphorylation of retinoblastoma (Rb) proteins, while Ccnd1 initiates the phosphorylation of Rb1 in mid-G1 phase [30, 31]. Rb1 physically interacts with Skp2 to inhibit Cdkn1b ubiquitination and induce G1 arrest [32]. Further, Anapc1 and its activator Cdh1 interact with Rb1 and are required for Rb1-induced cell cycle arrest which leads to Rb1-induced accumulation of P27 (Cdkn1b) during G1 arrest [33]. Detection of the Rb1 → Abl1 edge is illustrated in Fig. 4. Rb1 is known to form a complex with Abl1 in the late-G1/early-S-phase as a result of its hyperphosphorylation by the cyclin-D/cdk4–6 complex [34–36].

The Rb1 → Tfdp1 and Rbl1 → E2f1 edges are captured in the reconstruction of the network representing G1 phase in Fig. 4. It is widely accepted that Rb1 and Rbl1 genes negatively regulate the G1/S transition of the cell cycle and enable cell growth by targeting key transcription factors, including E2Fs and transcription factor DP subunits [37–39]. In addition, trans-activation by the E2f1-Tfdp1 heterodimers is known to be inhibited by the retinoblastoma protein family [40].

#### E2f1–4

In Fig. 4, E2f1 is seen to interact with Mcm3, Cdc6, Orc1, and Cdc45. The E2F transcription factor upregulates the transcription of Mcm3 gene in the late G1 phase [41, 42]. Besides the minichromosome maintenance complex (MCM) genes, Cdc6, ORC, and Cdc45 genes that are components of the pre-replication complex are well-known E2F-inducible genes during the late G1 and G1/S boundary in the cell cycle [43–46]. The Tfdp1 → E2f1 interaction is also detected; it is widely established that Tfdp1 interacts and forms a heterodimer with E2f1 to regulate the cell cycle progression from G1 to S phase [47–49].

#### Ccnd1/Cdk4

We note the Ccnd1-Cdkn2b and Cdk4-Cdkn1b interactions in Fig. 4. Cdkn2b can physically interact with and inhibit the activity of D-type cyclin dependent kinases and Cyclin D/CDK complexes while the Cip/Kip proteins, including Cdkn1a and Cdkn1b, can inhibit G1 CDKs such as Cdk4 [30, 50–52]. We also see the Ccnd1 → Rbl1 and Cdk4 → Rbl1 interactions in Fig. 4. It is known that in late G1 phase, Cyclin D/Cdk4–6 complexes perform the main phosphorylation of Rbl1, a member of the retinoblastoma family, leading to dissociation of Rbl1 from Rb-E2F/DP complexes [53–55]. Furthermore, the phosphorylation of Rbl1 by Cyclin D/Cdk4 complex inactivates Rbl1 to promote G1/S transition [55].

Ccnd1 → E2f1 and Ccnd1 → Tgfβ1 interactions are seen in Fig. 4. E2f1 is known to promote cell cycle progression through the induction of G1 phase cyclin, Cyclin D1 [56, 57]. Tgfβ1 blocks the progression of cell cycle during G1 and this is associated with Tgfβ1 inhibition of Ccnd1 expression [58]. We note the Ccnd1 → Cdh1 and Cdk4 → Cdh1 interactions; Cdh1 is known to limit the accumulation of the G1 mitotic cyclin/CDK complexes to prevent pre-mature S-phase entry [59]. Ccnd1 → Ccne1 is also captured in Fig. 4. Analyses by Geng et al. (1999) suggest that Cyclin E is a major downstream target of Cyclin D enabling cell to progress through G1 and enter the S phase [60].

#### Pre-replicative complex

The Orc1 ↔ Mamc3, Orc1 → Cdc6, Orc → Cdc7and Mcm3 → Orc1 interactions are also seen in Fig. 4. According to multiple studies, in late mitosis and during G1 phase, Orc1 bound to replication origins recruits and serves as a platform for the assembly of Cdc6 followed by Mcm3 to form the pre-replicative complex [61–64]. Orc1 interacts with Cdc6 throughout the G1 phase but not during other phases [62].

#### Kip/Cip cyclin dependent kinase inhibitors (Cdkn1a, Cdkn1b, and Cdkn2a)

The Cdkn1b → Tgfβ1, Mdm2 → Cdkn1a and Cdkn2a → Mdm2 regulatory links can be seen in Fig. 4. Tgfβ1 is reported to downregulate Cdkn1b during G1 phase [65] and Mdm2 has been shown to negatively regulate Cdkn1a and promote its proteasomal degradation which controls cell cycle progression during the G1 phase [66, 67]. Several studies have shown that Cdkn2a physically interacts with Mdm2 to impede Mdm2-induced degradation of Trp53 and enhances Trp53 role in transcription and apoptosis [68, 69]. This particular interaction stabilizes p53 and restores a p53-dependent G1 cell cycle arrest that is otherwise abrogated by MDM2 [52, 70, 71].

See supplementary text in Additional file 2 for extended description of interactions in the G1 phase.

### S phase

S (synthesis) phase is the second phase of the cell cycle occurring after the G1 phase and before the G2 phase in which DNA is replicated. Here we delve into the results for key S-phase proteins we obtained through our analysis (depicted in Fig. 5). Full list of the edges predicted for S phase is presented in Additional file 4.

#### Chek1

We note the Chek1 → Trp53 and Orc1 → Chek1 edges in Fig. 5. It is well established that Chek1 regulates Trp53 activity during DNA damage-induced S and G2 phase arrests [72–74]. Moreover, it has been extensively studied that cells with replicative initiation mutants defective in the Orc1 gene require the checkpoint kinase Chek1 during S phase to maintain cell viability by stabilizing DNA replication forks [75–77]. We note the interaction of Chek1 with Cdc45 and Cdk2 in Fig. 5. Cdc45 is a target of the Chek1-mediated S-phase checkpoint [78, 79]. During the S-phase checkpoint, Chek1 activity increases which leads to Cdk2 inhibition and blockage of the S-phase transit in response to DNA damage [80, 81]. We further note that Chek1 interacts with Smc1a and Wee1 in Fig. 5. Syljuåsen et al. (2005) have shown that inhibition of Chek1 in S-phase cells triggers rapid phosphorylation of Smc1a, suggesting a regulatory association between the two genes during S phase of the cell cycle to protect DNA breakage and promote DNA repair [79]. Chek1 phosphorylates and positively regulates Wee1 in the DNA replication checkpoint [82] and in the G2 DNA damage checkpoint [83]. Additionally, Wee1 inhibition diminishes Chek1 phosphorylation in cells that are undergoing replicative stress [84].

#### Atm

We note the E2f4 → Atm, Skp2 → Atm and Cdc7 → Atm edges in Fig. 5. E2F transcription factors not only regulate many genes required for entry into S phase, but also take part in DNA repair by transcriptionally regulating Atm [85]. Wu et al. (2012) have examined the role of Skp2 in DNA damage response and repair by showing its recruitment and activation of Atm during DNA double-strand breaks [86]. Cdc7, involved in initiation and progression of DNA replication during S phase, further plays role in DNA repair by activating the Atm/Atr-Chek1 checkpoint pathway [87].

#### Trp53

The interaction of Trp53 with Mcm3 and Orc1, both of which are key components of the pre-replicative complex, is shown in Fig. 5. Trp53 controls the initiation of replication and entry into S phase by regulating proliferation related genes such as Mcm3, Orc1, and Cdc6 [88, 89]. Furthermore, the Pkmyt1 → Trp53 interaction has been detected in the reconstruction of the S phase regulatory network. Price et al. (2002) have shown that Pkmyt1 can negatively regulate Trp53-induced apoptosis in response to DNA damage in the S phase or the G2 phase [90].

#### Mdm2

The Cdk1 → Mdm2 and Ttk → Mdm2 interactions can be seen in Fig. 5. Mdm2 is known to be phosphorylated by Cyclin A-Cdk1 complexes at the onset of S phase to reduce its interaction with Trp53 [91]. Ttk phosphorylates Mdm2 which facilitates oxidative DNA damage repair and cell survival during the S-phase [92].

#### Pre-replicative complex

We see the interaction of Mcm3 with Cdc45 in Fig. 5. Mcm3 and Cdc45, both interacting components of the pre-replicative complex [93–95], are known to dissociate from the origin DNA and associate with non-origin DNA and move with replication forks at the beginning of S phase [96, 97]. In addition, Cdc45 loading onto the chromatin in the S phase is required to activate the helicase activity of the MCM complex [98, 99]. We note the Cdc6 → Cdk2 edge; Cdc6 has been shown to activate Cdk2 to initiate DNA replication and G1-S phase progression [100, 101]. Cdc6 is known to activate Cdk2 to prevent re-replication during S and G2 phases [102]. Dbf4 → Cdk1 can be seen in Fig. 5; Cdk1 is known to target the Dbf4-Cdc7 kinase at the end of S phase to prevent re-replication in G2/M [103, 104].

Further description of S phase-specific interactions is provided in supplementary text in Additional file 2.

### G2/M phase

G2 phase is the third phase of the cell cycle in which the cell rapidly grows, protein synthesis occurs, and the cell prepares to enter mitosis. During mitosis, the replicated chromosomes are separated into two nuclei and the cell is divided into two daughter cells. Additional file 5 consists of the entire list of interactions estimated in reconstruction of the G2/M phases. In this section, we investigate the main G2/M signaling pathways predicted in our study (shown in Fig. 6).

#### Ttk

We note the Ttk → Bub1, Ttk → Mad2l1, and Ttk → Bub1b interactions in Fig. 6. Studies have revealed that Mph1 (Ttk homologue), which localizes to the kinetochores only at prometaphase (second phase of mitosis), is required for the recruitment of Bub1 and other spindle assembly checkpoint components [105, 106]. Ttk promotes closed Mad2l1 production and subsequent assembly of the mitotic checkpoint complex (MCC) to activate the spindle checkpoint assembly [107]. Huang et al. (2008) have reported that Ttk is one of the major kinases required for Bub1b phosphorylation which is essential for the mitotic checkpoint and also for kinetochores to establish microtubule attachments during G2/M [108].

#### Mad2l1-Mad1l1

The Espl1 → Mad2l1, Mad2l1 → Bub1b, Bub3 → Mad1l1, and Rad21 → Mad1l1 edges can be seen in Fig. 6. The Espl1-Mad2l1 interaction has been confirmed as a regulatory mechanism required for sister chromatid segregation [109]. Further, the spindle assembly checkpoint components Mad2l1 and Bub1b are known to act cooperatively to assemble the mitotic checkpoint complex and to prevent premature chromatid separation at the mitotic checkpoint [110–112]. Multiple studies have indicated that Mad1l1 forms a complex with Bub3 during the cell cycle and is crucial for spindle checkpoint function [113–115]. There is evidence that knockdown of MAD proteins is correlated with Rad21 cleavage to promote sister chromatid segregation [116].

#### Bub1b-Bub1-Bub3

The Bub1b → Cdc20 and Bub1b → Plk1 edges can be seen in Fig. 6. Studies have shown that a checkpoint function of Bub1b is to inhibit the activity of Anaphase Promoting Complex (APC/C) by blocking the binding of Cdc20 to APC/C [117–119]. Furthermore, Bub1b binds to Cdc20 to inhibit APC activity in interphase, allowing the accumulation of Cyclin B in G2 phase prior to M-phase entry [120]. Bub1b localizes to centrosomes and suppresses centrosome amplification via regulating Plk1 activity during interphase [121]. In addition, Bub1b brings about the action of Plk1 at kinetochores for appropriate chromosome alignment during prometaphase [122].

#### Cdk1

We see the interaction of Cdk1 with Bub1b and Rbl1 in Fig. 6. Phosphorylation of Bub1b by Cdk1 is required for mitotic spindle checkpoint arrest and promotes the formation of the kinetochore during G2/M [123]. It has been reported that Cdk1 phosphorylates pRB (retinoblastoma protein) in mitotic cells [36, 124, 125], while our model captures the interaction of Cdk1 with the pRB-related protein, Rbl1.

The network of Fig. 6 depicts the edges Cdk1 → Ccnb2, Wee1 → Cdk1, and Pkmyt1 → Cdk1. B-type cyclins form a complex with Cdk1 and this complex accumulates through late S and G2 phases of the cell cycle [126] and the activation of the Cyclin B-Cdk1 kinase is needed for entry into the G2/M phase [127, 128]. It is widely accepted that Cdk1 activity is regulated through its inhibitory phosphorylation by Wee1 and Pkmyt1, leading to activation of the G2/M arrest which prevents premature entry into mitosis [129–132].

#### Ccnb2

We can see the Cdc20 → Ccnb2 and Cdc25b → Ccnb2 edges in Fig. 6. It is known that APC/C-Cdc20 interaction can mediate cyclin B degradation which consequently prevents Cdk1 activity from reaching excessively high levels [133] and that the spindle assembly checkpoint acts on Cdc20 to block the degradation of Cyclin B during metaphase [134]. The Cdc25 phosphatases are known to dephosphorylate and therefore activate the Cdk1-Cyclin B complexes [135–137].

#### Espl1

The Espl1 → Ccnb2, Cdk1 → Espl1, Espl1 → Smc1a, and Espl1-Bub1 interactions are shown in Fig. 6. Espl1 binds to Cyclin B during anaphase, a required step in anaphase to shut down Cdk1 activity, to achieve abrupt and simultaneous separation of sister chromatids [138–140]. It is widely accepted that Espl1 triggers anaphase (fourth phase of mitosis) by initiating cleavage of cohesin multiprotein complex which includes the Smc1a subunit [141]. Studies have determined the role of Bub1 in the timing of Espl1 activation and hence regulation of anaphase [142, 143]. The supplementary text in Additional file 2 provides an extended description of G2/M phase-specific network identified in our study.

## Discussion

Mammalian cell cycle is a dynamic process orchestrated by the activation of distinct molecular players across time. Canonical characterization of the cell cycle as a static network fails to provide temporal mechanistic insights on the control exerted by the proteins during different phases of the cycle. In this study, we use an exhaustive and fine-grained time series expression dataset capturing the cell cycle of MEF primary cells to develop a temporally evolving dynamical network for the cell cycle progression. While our reconstruction is based on using the transcriptome, and there could be differences between the transcriptome and the proteome abundances [144–147], we believe that the broad conclusions are substantiated by mechanisms reported in the literature. For example, studies have revealed that certain classes of genes, such as cell cycle genes, have higher correlation of mRNA expression with the corresponding protein expression across a large number of genes [148, 149], justifying our use of the transcriptome across time to investigate the cell cycle. Using a set of 63 key cell cycle genes, we show that our causality-driven approach provides a temporal map of the phases of the cell cycle.

The mechanistic changes in the RNA-seq time-course data are identified by a change point detection algorithm which enables us to infer the timing of cell cycle phases and their duration with no prior biological knowledge. Through our computational analysis, the G1, S, and G2/M phases are estimated to be 14.5 h, 10 h, and 4 h long, respectively. For a typical proliferating mammalian cell with an average cycle span of 24 h, G1 phase lasts about 11 h, S phase about 8 h, G2 phase about 3–4 h, and M phase about 1 hour [150]. However, cell cycle duration varies from one cell type to another; for instance, the average phase duration for the rat embryo PC12 cell line when serum starved for 24 h and then serum treated for 37 h, is roughly 15 h, 13.3 h, and 4 h for the G1, S, and G2/M phases, respectively [151], whereas reports show that the average cell cycle length for MEF cell line is 25.3 h [152, 153].

The three successive directed graphs depicted in Figs. 4, 5, and 6, representing the interaction of cell cycle genes as the cell evolves through the G1, S and G2/M phases of the cell cycle, are derived by utilizing the notion of Granger causality identified by a VAR model. This approach allows for the inference of temporal length of influences each gene has on others. The temporal dependencies are obtained by estimating the optimal order of the VAR model that reveals the sufficient number of lags required to extract useful past information that may influence the expression of other genes.

Among the key G1 phase mechanisms (Fig. 4), we were able to detect the regulation of Rbl1 by Ccnd1 and Cdk4 as a promoting factor in the G1/S transition [55], the role of the retinoblastoma protein in enabling cell growth by targeting E2F and DP transcription factors [37–39], as well as the function of cyclin dependent kinase inhibitors in inducing growth arrest in the G1 phase [51, 52]. The Cdkn2a-Mdm2 interaction which stabilizes the tumor suppressor protein Trp53 [71], the Cyclin E-Cdk2 interaction required for G1/S transition [154], as well as Ccne1’s role in the loading of Mcm3 and Cdc45 onto the chromatin [101, 155, 156] were detected in our reconstruction of the G1 phase network. We were able to detect the recruitment and assembly of Mcm3 and Cdc6 by Orc1 leading to the formation of the pre-replication complex and its assembly onto replication origins prior to S phase [61, 63].

The G1 phase events prepare the cell to initiate DNA replication in the S phase of the cell cycle. The pre-replicative complex is assembled onto each origin prior to S phase and creates licensed origins that can initiate replication by origin firing. Once the cell transitions from G1 phase to the S phase, the licensed origin are converted into active replication forks [157, 158].

Major S-phase regulatory pathways are shown in Fig. 5. The loading of the replicative polymerases through Mcm3 recruitment of Cdc45 [159], along with the intra S-phase checkpoint exerted by Chek1 targeting of Cdc45 and regulation of Cdk2 [78, 80], are among the major S-phase pathways. The network in Fig. 5 further describes the role of Chek1 in stabilizing the replication forks and protecting against DNA breakage through its interaction with Orc1 and Smc1a [76, 79].

During S-phase, Trp53 is involved in regulating initiation of replication by targeting replication-related genes Cdc6, Orc1, and Mcm3 [88]. Furthermore, we detected Cdk1’s role in preventing re-replication during S phase by regulating Dbf4 [104], along with the function of Atm in regulation of DNA damage and DNA repair, captured through Atm’s interaction with Dbf4 and Skp2 [85, 86].

Figure 6 represents significant regulatory pathways characterized in the G2/M phases of the cell cycle such as spindle assembly checkpoint (SAC), mitotic checkpoint assembly, and chromosome segregation. Among these pathways, we detected the formation of mitotic checkpoint complex and the establishment of microtubule attachments during G2/M phases through the function of Ttk-Mad2l1 and Ttk-Bub1 interactions, respectively [106, 108]. Our proposed model for the G2/M phase identifies the Bub3-Mad1l1 interaction essential for spindle checkpoint function [113–115], the cooperative interaction of Mad2l1 and Bub1b that is required for prevention of premature sister chromatid segregation [111], as well as Mad2l1-Chek1 interaction which ensures fidelity of mitotic segregation [160]. In addition, we detected the interactions suggesting the blockage of premature entry into mitosis through Cdk1 phosphorylation by Wee1 and Pkmyt1 [129–132]. It is interesting that Cdk1 phosphorylation not only occurs at an early G2 phase, but may also occur during late S phase [132] as shown in Figs. 5 and 6. We detected Cdk1’s role in preventing re-replication during G2/M by targeting Cdc7 [103], along with the activation of the Cdk1-Cyclin B complex required for G2/M entry [127]. Additionally, we spotted Plk regulation of Cdc20 which activates the anaphase promoting complex, triggering the separation of sister chromatids [161], Plk1’s role in mitotic exit through its interaction with Cdc25b [162], as well as the concurrent and abrupt segregation of sister chromatids through the Espl1-Ccnb2-Cdk1 pathway [138, 140] (Fig. 6).

We also built the above networks by incorporating transcription factors (TFs) in our analysis using TRANSFAC® (2018, release 2) database. We found 25 differentially expressed TFs in our dataset that we considered in this analysis. There is significant overlap between the intra-cell cycle gene interactions when comparing the results from the 63 cell cycle genes-based network model and that from the 63 cell cycle genes- and 25 TFs-based network model. The list of all interactions for the TFs and cell cycle genes for the G1, S and G2/M phases is included in Additional file 6. We validated our inference through STRING database for intra-cell cycle interactions, and through Enrichr libraries [163, 164] for those involving TFs. We also calculated the precision metric for the network reconstructed using the dataset incorporating TFs. Additional file 2: Table S2 shows the precision metric for the three networks.

## Conclusions

In this work, we reconstruct causal mechanisms and networks across time during a mammalian cell cycle. Our integrative framework provides insight into the temporal behavior of MEF cell cycle. Through our computational analysis of the time series data from cell cycle genes, without any prior knowledge, we estimate the G1, S and G2/M phases of the cell cycle to last approximately 14.5, 10 and 4 h, respectively. Using the data from each cell cycle phase, we reconstruct phase specific networks, and detect key regulatory interactions essential to passage of the cell through cell cycle checkpoints. Since we utilize a higher order VAR model, the delay between the synthesis of the transcript and its corresponding protein is taken into account, justifying our use of temporal gene expression data in our analysis. Moreover, the average optimal order for genes participating in each regulatory pathway is used as a means to determine the temporal dependencies between multiple biological pathways in the three successive cell cycle regimes that transcends the current understanding of temporal interdependency of cellular mechanisms in the biology literature.

## Methods

### RNA-seq data

The gene expression profiles are acquired through a RNA-seq experiment for serum response of Cf-1 MEF primary cells (E13 embryos), the purpose being to transcriptionally characterize the changes in the cell cycle genes as the cell cycle progresses. In order to synchronize the cell cycle, after the cells are incubated in starvation medium (0.5% FCS) for 36 h, serum is added to reach 20%, to re-initiate the cell cycle. RNA isolation is performed using Trizol RNA extraction protocol. The RNA-seq data is aligned using the STAR RNA-seq aligner [165] and the read counts are normalized using the HOMER software [166]. Samples are taken 1 hour before the addition of serum, right before the addition of serum and every half hour after serum addition. This sampling routine is carried out for approximately two cell cycles. The raw time-series data is then processed to determine the fold-change in expression for each gene by dividing the expression level of each sample by the average of the expression levels at samples taken 1 hour before serum addition and right before serum addition.

### Change point detection algorithm

Change Point Detection (CPD) is a non-parametric method based on sequential application of Singular Spectrum Analysis (SSA) to detect changes in time-series [22, 167]. SSA is a powerful method for time-series analysis that is based on applying principal component analysis to the trajectory matrix acquired from the original time series. Basic SSA has four main steps:

1. Embedding: In this step, the trajectory matrix *X* is built from the original time series, *x*_*t*_, where every column of this matrix is a vector that lies in an *M*-dimensional space *ℝ*^*M*^.
2. Singular value decomposition (SVD): The singular value decomposition of the lag-covariance matrix *R* = *XX*^*T*^ provides *M* eigenvalues and eigenvectors and principal components.
3. Grouping: The principal components are split into two groups *I* and *I*′. The *l* principal components in group *I* provide a good description of the time series. This leads to decomposition of the trajectory matrix $X={X}_I+{X}_{I^{\prime }}$.
4. Diagonal averaging: Through this step, the matrices *X*_*I*_ and ${X}_{I^{\prime }}$ are uniquely mapped to their respective time-series *z*_*t*_ and *ε*_*t*_.

**Fig. 9:**
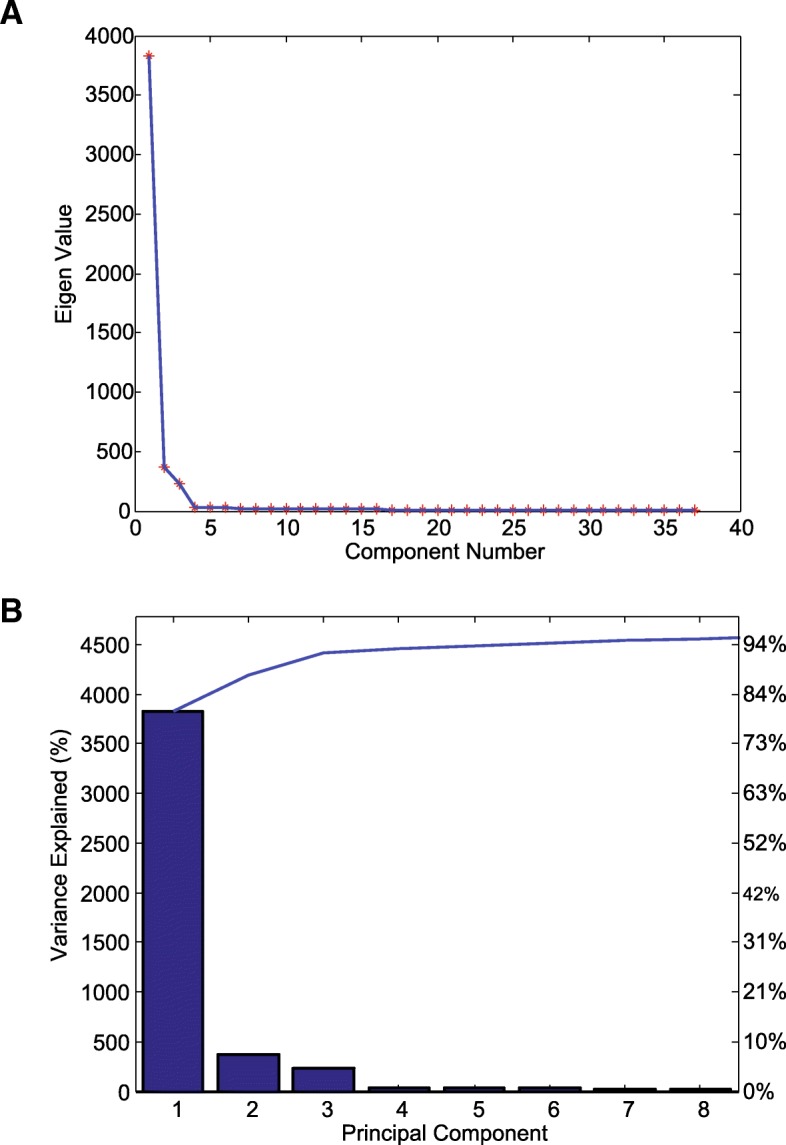
Principal Components of Cdkn2d gene expression profile. **a** Scree plot of the Eigenvalues shows the ordered eigenvalues of the lag-covariance matrix corresponding to the gene expression profile of Cdkn2d. We can see a dramatic change in slope of the eigenvalue plot at the fourth component. Therefore, from what is observed in this plot, it is reasonable to retain the first three largest eigenvalues and group them together to select the set *I*. **b** Explained variance for the first eight largest principal components that explain 95% of the cumulative variation. The fourth and higher components explain very little variation and thus the first three largest eigenvalues can be grouped together in group *I*

The SSA captures the structure of the time-series by selecting the *l* eigen-vectors, which span an *l*-dimensional subspace. Figure 9a and b show the scree plot and explained variance of eigenvalues, respectively when SVD is applied to the time-course data of Cdkn2d.

**Fig. 10:**
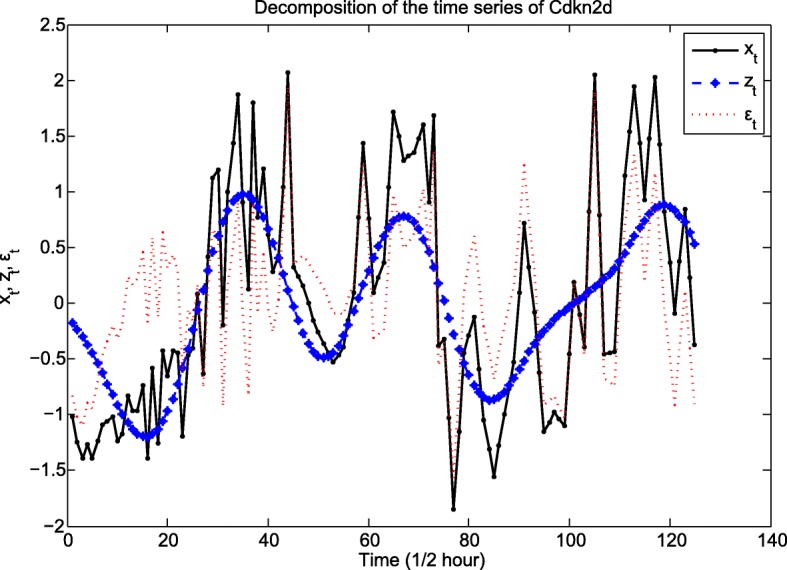
Decomposition of Cdkn2d time series into the main signal and noise. This plot depicts the time series *x*_*t*_ for the gene expression profile of Cdkn2d (black curve), along with its decomposition into two time series *z*_*t*_ and *ε*_*t*_. *z*_*t*_ (blue curve) corresponds to the time series reconstruction from matrix *X*_*I*_ that is built from the three largest eigenvalues of the lag-covariance matrix, and *ε*_*t*_ (red dotted curve) corresponds to the time series reconstruction from matrix ${X}_{I^{\prime }}$ that is built from the remaining eigenvalues of the lag-covariance matrix

**Fig. 11:**
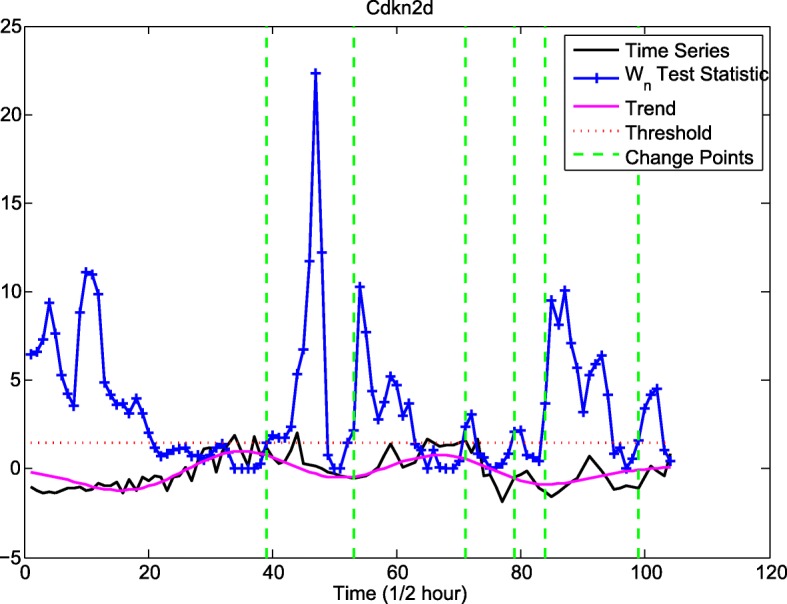
Plot of change points for Cdkn2d time series. The black curve is the original RNA-seq time series data for Cdkn2d, *x*_*t*_. The pink curve corresponds to *z*_*t*_ which is the trend of the time series. The blue curve is the plot of the *W*_*n*_ test statistic calculated through the CPD algorithm. The dotted red curve is the threshold *h* which is used in the decision rule. Every time the *W*_*n*_ test statistic exceeds the threshold, a change point is selected (green dotted lines)

This helps choose the number of eigenvalues that capture sufficient variation in the time series (see the trend of Cdkn2d time-series displayed by the three largest eigenvalues in Fig. 10). Change point detection can be achieved by sequentially applying SVD to the lag-covariance matrices computed in time intervals of length *N*, [*n* + 1,  *n* + *N*], and selecting a group of *l* eigenvectors determining an *l*-dimensional subspace. If at a particular time *τ*, an increase in the distance between the *l*-dimensional hyperplane spanned by the eigenvectors of the lag-covariance matrix and the M-lagged vectors *X*_*j*_ (*x*_*j* + 1_,  … , *x*_*j* + *M*_), *j* ≥ *τ*, occurs, the algorithm postulates a change point. A cumulative sum-type statistic is computed based on the distance between the *l*-dimensional subspace and the vectors *X*_*j*_ and compared against a threshold; every time the test statistic exceeds the threshold, a change point is detected. Figure 11 depicts the detection of change points in the time-series for Cdkn2d time-series. The change points detected are representative of a structural change in the mechanism generating the time-series (see the supplementary methods in Additional file 2).

### Granger causality

Granger causality is a notion based on the ability to predict the future value of one process using the past values of another process [25]. This notion was first introduced in macroeconomics and has proven useful in providing the direction of information flow; however it is not equivalent to true causality. Granger causality provides information about numerical information and prediction, while true causality is profoundly related to the influence of one variable onto another. Formally, a time series *x* is said to Granger-cause a time series *y* if the future value of *y* can be better predicted given the past values of *x* and *y*, (*x*_*t* − 1_, *x*_*t* − 2_, … , *y*_*t* − 1_, *y*_*t* − 2_, … ), than predicting the future of *y*_*t*_ given only the past values itself, (*y*_*t* − 1_, *y*_*t* − 2_, … ). This statistical concept of causality can be well represented by the VAR model for linear relationships [24]. A *d*-order VAR model of a *k* dimensional time series is given by:

3 $$ y(t)=v+{A}_1y\left(t-1\right)+{A}_2y\left(t-2\right)+\dots +{A}_dy\left(t-d\right)+\varepsilon (t) $$

where *y*(*t*) = (*y*_1_(*t*), *y*_2_(*t*),  … , *y*_*k*_(*t*))^*T*^ is a (*k* × 1) random vector, *y*_*i*_(*t*) is the measurement at time *t* of the *i*^*th*^ random variable, *A*_*l*_ is a (*k* × *k*) autoregressive coefficient matrix, ***v*** is a (*k* × 1) vector of intercepts and *ε*(*t*) = (*ε*_1_(*t*), *ε*_2_(*t*),  … , *ε*_*k*_(*t*))^*T*^ is a *k*-dimensional error vector of random variables with zero mean and covariance matrix ∑.

A necessary and sufficient condition for variable *y*_*j*_ to be Granger-causal for *y*_*i*_ is that the corresponding coefficient *a*_*ijl*_ (*ij*^*th*^ entry of *A*_*l*_, *l* = 1, … , *d*) is statistically significant [168, 169]. Therefore, the direction of information flow can be determined by estimating the autoregressive coefficient matrices of the VAR model. The optimal order of the VAR model can be estimated via the minimum description length (MDL) principle.

### Estimation stability with cross validation

Considering the time series (*y*_1_, … , *y*_*T*_) for each of the *k* variables, the VAR model in Eq. (3) can be written compactly in the following matrix form [170]:

4 $$ Y=\varphi X+\varepsilon $$

where *Y* = (*y*_1_,  … , *y*_*T*_)^*T*^ is a (*T* × *k*) matrix whose columns are time series for each of the *k* random variables with sample size *T*, *φ* = (*φ*_0_,  … , *φ*_*T* − 1_)^*T*^ is a (*T* × (*kd* + 1)) matrix with *φ*_*t*_ = (1; *y*(*t*);  … ; *y*(*t* − *d* + 1))^*T*^, *X* = (*v*, *A*_1_, *A*_2_,  … , *A*_*d*_)^*T*^ is a ((*kd* + 1) × *k*) coefficient matrix and *ε* = (*ε*_1_,  … , *ε*_*T*_)^*T*^ is a (*T* × *k*) matrix. For each of the *k* columns of matrices *Y*, *X*, and *ε*, we have the following linear regression model

5 $$ {y}_i=\varphi {x}_i+{\varepsilon}_i,\kern0.5em i=1,\dots, k $$

We are interested in recovering vector *x*_*i*_ ∈ *ℝ*^*kd* + 1^ from the observation *y*_*i*_ ∈ *ℝ*^*T*^ and *φ*. Since *φ* ∈*ℝ*^*T* × (*kd* + 1)^, and *T* ≪ *kd* + 1, we have an underdetermined system of linear equations, and this linear inverse problem cannot be solved uniquely. However, if *x*_*i*_ is sufficiently sparse, i.e., the support of *x*_*i*_ has small cardinality, it is actually possible to recover *x*_*i*_ by solving the following *ℓ*_0_ minimization problem [171, 172]:

6 $$ {\widehat{x}}_i=\min \left\Vert {x}_i\right\Vert {}_0\kern0.75em subject\ to\kern0.5em {y}_i=\varphi {x}_i,\kern0.5em i=1,\dots, k $$

where ‖*x*_*i*_‖_0_ denotes the number of nonzero coefficients of *x*_*i*_. Since *ℓ*_0_ minimization is an NP-hard problem, it can be relaxed to an *ℓ*_1_-norm regularization that can be a heuristic for finding a unique sparse solution [173]:

7 $$ {\widehat{x}}_i=\min\ {\left\Vert {y}_i-\varphi {x}_i\right\Vert}_2+\lambda\ {\left\Vert {x}_i\right\Vert}_1\kern0.5em ,\kern0.5em i=1,\dots, k $$

Note that *ℓ*_1_-norm regularization in Eq. (6) is strictly related to the Least Absolute Shrinkage and Selection Operator (LASSO) problem [174]:

8 $$ {\widehat{x}}_i=\min \frac{1}{2}{\left\Vert {y}_i-\varphi {x}_i\right\Vert}_2^2+\lambda\ {\left\Vert {x}_i\right\Vert}_1,\kern0.75em i=1,\dots, k $$

The regularization parameter *λ* in the LASSO sets a trade-off between the fit error ${\left\Vert {y}_i-\varphi {x}_i\right\Vert}_2^2$ and the sparsity of the signal *x*_*i*_. In order to choose the desired *λ*, one can use traditional model selection criteria, such as Akaike’s information criterion (*AIC*) [175] and Bayesian information criterion (*BIC*) [176]. These criteria are easily computed, though are dependent on model assumptions and even if model assumptions are met, they may not be valid in the finite sample cases. The regularization parameter *λ* is often selected through the model-free Cross-validation (CV) approach [177, 178]. CV often leads to estimators with good predictive performance when sample size is large. In the cases where sample size is small, CV does not yield a good interpretable model because LASSO + CV is unstable and not reliable for scientific interpretations [179]. In this work, we observed that selecting *λ* through *Estimation Stability with Cross Validation* (ES-CV) leads to more meaningful and interpretable results [180]. Estimation stability (ES) is based on the idea that the solution is not meaningful if it varies considerably from sample to sample. The LASSO generates a family of solutions known as the solution path:

9 $$ {\widehat{x}}_i\left[\lambda \right]=\mathit{\operatorname{minimize}}\ {\left\Vert {y}_i-\varphi {x}_i\right\Vert}_2^2+\uplambda {\left\Vert {x}_i\right\Vert}_1 $$

We want to choose *λ* in the solution path based on estimation stability. Since ES is tightly tied to the sampling scheme, we need multiple solution paths to evaluate stability. Cross-validation data perturbation is used to randomly partition the *T* samples into *V* groups of pseudo data sets by leaving out one group at a time. Let *φ*^∗^[*j*], *y*_*i*_^∗^[*j*] represent the *j*^*th*^ pseudo data set (random partition) derived from *φ* and *y*_*i*_, respectively. The pseudo solutions are given by:

10 $$ {\widehat{x}}_i\left[j;\lambda \right]=\mathit{\operatorname{minimize}}{\left\Vert {y_i}^{\ast}\left[j\right]-{\varphi}^{\ast}\left[j\right]{x}_i\right\Vert}_2^2+\lambda {\left\Vert {x}_i\right\Vert}_1 $$

for *j* = 1, … , *V*, *i* = 1, … , *k*. ES measures the stability or similarity of pseudo solutions across different groups of samples. For each *λ*, the stability of the following estimates

11 $$ {\widehat{y}}_i\left[j;\lambda \right]=\varphi {\widehat{x}}_i\left[j;\lambda \right],\kern0.5em j=1,\dots, V,\kern0.5em i=1,\dots, k $$

are studied by looking at the sample variance of the estimates

12 $$ \widehat{VAR}\left({\widehat{y}}_i\left[\lambda \right]\right)=\frac{1}{V}{\sum}_{j=1}^V{\left\Vert {\widehat{y}}_i\left[j;\lambda \right]-{\overline{\widehat{y}}}_i\left[\lambda \right]\right\Vert}_2^2,\kern0.75em j=1,\dots, V,\kern0.5em i=1,\dots, k $$

where

$$ {\overline{\widehat{y}}}_i\left[\lambda \right]=\frac{1}{V}{\sum}_{j=1}^V{\widehat{y}}_i\left[j;\lambda \right]. $$

The normalized version of the sample variance is defined as the estimation stability metric:

13 $$ ES\left(\lambda \right)=\frac{\widehat{VAR}\left({\widehat{y}}_i\left[\lambda \right]\right)}{{\left\Vert {\overline{\widehat{y}}}_i\left[\lambda \right]\right\Vert}_2^2} $$

*ES* is the reciprocal of the test statistic for testing the null hypothesis *H*_0_ : *φx*_*i*_ = 0, and can be viewed as a selection of *λ* as a set of hypothesis tests; for each *λ* we are testing to see if the fit ${\widehat{y}}_i\left[\lambda \right]$ is statistically different from fitting the null model (*φx*_*i*_ = 0).

**Fig. 12:**
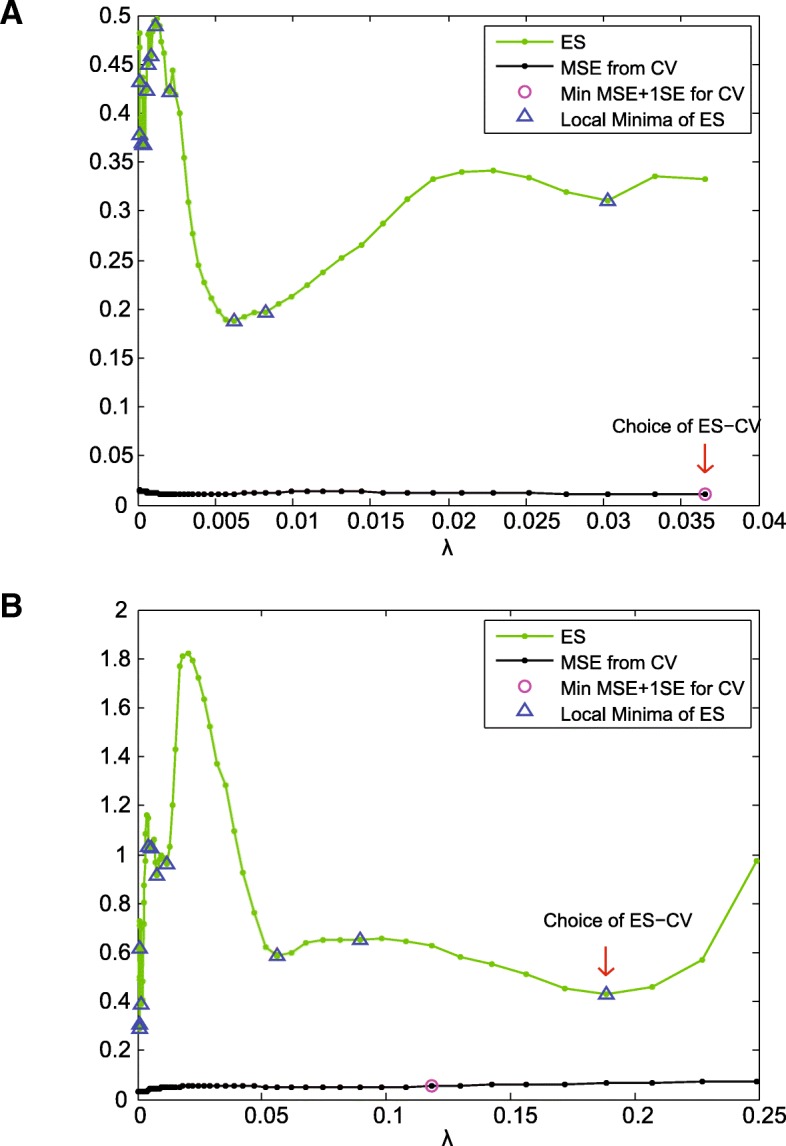
Estimation Stability with Cross Validation (ES-CV). The green curves are the plot of the ES metric. The black curves are the plot of mean squared error (MSE) through cross validation. The blue triangles identify the *λ* at which the local minima of the ES metric occur. The pink circles indicate the largest *λ* such that MSE is within one standard error of the minimum MSE. **a** In the case where noise overwhelms the data, ES fails and CV is incorporated. We can note that between the choice of CV (pink circle) and the choice of ES (blue triangles), ES-CV picks the larger *λ*. **b** We can note that the ES-CV approach selects a larger *λ* compared to the choice of cross validation. Hence, the choice of *λ* selected through ES-CV leads to a sparser solution than that of CV

The most statistically significant solution along the solution path is the one whose ES metric has the largest reciprocal. Therefore, the most statistically significant solution is the one that locally minimizes the *ES* metric. In the case where noise overwhelms the signal (high noise), *y* bears no relation to *φ* and *ES* proposes inadvertent local minima. Thus, cross-validation is incorporated into finding the solution (ES-CV) (see Fig. 12a). ES-CV further limits the choice of *λ* to the local minimum of *ES*(*λ*) that is greater than or equal to the choice of cross-validation (see Fig. 12b) [179, 180].

### Minimum description length

The optimal order of the VAR model can be estimated through model selection approaches such as Minimum Description Length (MDL) [26]. MDL selects a model that provides the shortest description of data. Description length for observations *y*^*T*^ = {*y*_1_, *y*_2_,  … , *y*_*T*_} from a parametric family $\mathcal{M}=\left\{f\left({y}^T|\theta \right):\theta \in \Theta \right\}$ is $-\mathit{\log}{f}_{\theta}\left({y}^T\right)+\mathcal{L}\left(\theta \right)$, where the first term is the cost function and the second term is the cost of transmitting the estimated parameter *θ*. For a linear regression model in eq. (3), the observation *y* has the following description length:

14 $$ DL=\frac{T}{2}\log RSS+\frac{d}{2}\log T;\kern0.5em d=1,2,\dots, {d}_{max} $$

where *RSS* denotes the residual sum of squares and *d* is the order of the VAR model in Eq. 3. The optimal order is selected such that the code length in Eq. 14 is minimized:

15 $$ {d}_{opt}=\mathit{\operatorname{minimize}}\ \frac{T}{2}\log RSS+\frac{d}{2}\log T;\kern0.5em d=1,2,\dots, {d}_{max} $$

### Precision

Precision or confidence indicates the proportion of predicted positive edges that are real positives [181]. In other words, Precision is a measure of accuracy of the predicted positives:

$$ Precision=\frac{True\ Positives}{True\ Positives+ False\ Positives} $$

These methods have been implemented in Matlab® (Mathworks, Inc.).

## Additional files

Additional file 1: List of 63 cell cycle genes and their full names. (XLSX 11 kb)

Additional file 2: Supplementary methods and supplementary text. (DOCX 64 kb)

Additional file 3: Complete list of interactions identified in reconstruction of G1 phase in the {1–14.5} hour interval. (XLSX 23 kb)

Additional file 4: Complete list of interactions identified in reconstruction of G1 phase followed by S phase in the {1–24.5} hour interval. (XLSX 21 kb)

Additional file 5: Complete list of interactions identified in reconstruction of the G1 and S phases followed by G2/M phases in the {1–28.5} hour interval. (XLSX 21 kb)

Additional file 6: Complete list of interactions identified in reconstruction of the three networks incorporating transcription factors using data from the {1–14.5} hour, {1–24.5} hour and {1–28.5} hour intervals. (XLSX 25 kb)

## Abbreviations

AIC: Akaike’s Information Criterion
BIC: Bayesian Information Criterion
CPD: Change Point Detection
CV: Cross Validation
ES-CV: Estimation Stability with Cross Validation
KEGG: Kyoto Encyclopedia of Genes and Genomes
LASSO: Least Absolute Shrinkage and Selection Operator
MDL: Minimum Description Length
MEF: Mouse Embryonic Fibroblast
PCR: Principal Component Regression
PLS: Partial Least Squares
SSA: Singular Spectrum Analysis
SVD: Singular Value Decomposition
VAR: Vector Autoregressive
