# Supplementary material for: Time varying causal network reconstruction of a mouse cell cycle
Source: BMC Bioinformatics. 2019 May 29;20:294. doi: 10.1186/s12859-019-2895-1 (PMC6542064; doi:10.1186/s12859-019-2895-1)
Supplement: Supplementary file 2 — Supplementary methods and supplementary text. (DOCX 64 kb) [file 12859_2019_2895_MOESM2_ESM.docx]

Additional File for

Time Varying Causal Network Reconstruction of a Mouse Cell Cycle

Maryam Masnadi-Shirazi, Mano R. Maurya, Gerald Pao, Eugene Ke, Inder M. Verma and Shankar Subramaniam

Correspondence to: Shankar Subramaniam. E-mail: shankar@ucsd.edu

**This PDF file includes:**

Supplementary Methods

Supplementary Text

Supplementary Tables S1-S2

Supplementary References

Supplementary Methods

Evaluating association between the time-series of two cell cycles

The RNA-seq experiment was done for two cell cycles (for mouse embryonic fibroblast primary cells) following serum starvation and the addition of serum. Serum starvation and refeeding for mammalian cell division does not necessarily result in synchronization of the entire cell population [[1](#_ENREF_1), [2](#_ENREF_2)]. Thus, the two time-series data acquired through RNA-seq does not reflect the behavior of synchronized cells, and therefore they may not have begun at the same occasion of measurements. The time interval separating the start of the two cell cycles is called *delay* or *offset*. A common approach to finding the association between events in two time-series is cross-correlation in which the Pearson product moment correlation is computed for the two time-series [[3](#_ENREF_3)]. The offset is determined by finding the sample at which the highest cross-correlation between the two time-series occurs. Fig. 1 shows the plot of the cross correlation of the two available time-series for Smc1a gene.

Change point detection algorithm

Here we present a detailed description of the change point detection algorithm that was utilized in detecting cell cycle checkpoints in this work. The main idea of this model-free algorithm is built on the basis of singular spectrum analysis (SSA) [[4](#_ENREF_4)]. Basic SSA has the following four steps:

1. **Embedding**

Let $x_{1}, x_{2}, \ldots, x_{T}$ be a time series of length $T$, $M(M\leq T/2)$ be some integer called ‘lag’, and let $K=T-M+1$. Define the trajectory matrix

$X={(x_{ij})}_{ij=1}^{M,K}=\left( \begin{matrix} x_{1} & x_{2} & x_{3} & \ldots& x_{K} \\ x_{2} & x_{3} & x_{4} & \ldots& x_{K+1} \\ \vdots& \vdots& \vdots& \ddots& \vdots\\ x_{M} & x_{M+1} & x_{M+2} & \ldots& x_{T} \end{matrix} \right)$ (1)

Note that the columns of the trajectory matrix $X_{j} (j=1, \ldots, K)$ are vectors that lie in an $M$-dimensional space $\mathbb{R}^{M}$space.

1. **Singular Value Decomposition**

Let $R=XX^{T}$ be the lag-covariance matrix. The Singular Value decomposition (SVD) of $R$ provides us with $M$ eigenvalues, eigenvectors and principal components. $\gamma_{1}, \gamma_{2}, {\ldots, \gamma}_{M}$ denote the eigenvalues of $R$ and $U_{1}, U_{2}, \ldots, U_{M}$ are the corresponding orthonormal eigenvectors of $R$. If $b$ is the number of non-zero eigenvalues, and $V_{i}$ the eigenvector of $X^{T}X$, we have $V_{i}=X^{T}U_{i}$ for $i=1, \ldots, b$. Then SVD of X will yield $X=X_{1}+X_{2},+\ldots, X_{b}$, where $X_{i}=\sqrt{\gamma_{i}}U_{i}V_{i};i=1, .., b$.

1. **Grouping** The indices $\left\{ 1, 2, \ldots, b \right\}$ can be split into two groups $I=\{i_{1}, \ldots, i_{l}\}$ and $I'=\left\{ 1, \ldots, b \right\}\backslash I$. Matrices $X_{I}=\sum_{i\in I} X_{i}$ and $X_{I^{'}}=\sum_{i\notin I} X_{i}$ correspond to group $I$ and $I'$ and lead to the decomposition $X=X_{I}+X_{I^{'}}$.
2. **Diagonal Averaging (Hankelization)**

This step transforms each matrix of the grouped decomposition in the previous step into new time series of length $T$ and is performed by averaging the diagonals $i+j=const$ of the Hankel matrices, $X_{I}$ and $X_{I^{'}}$. Hankelization is an optimal procedure that uniquely defines the one-to-one correspondences between the Hankel matrices $X_{I}$ and $X_{I'}$ and their respective time-series $z_{t}$ and $\varepsilon_{t}$ of length $N$, leading to the decomposition of series $x_{t}$ into two series $z_{t}$ and $\varepsilon_{t}$

$x_{t}=z_{t}+\varepsilon_{t}.$ (2)

$z_{t}$ and the residual series $\varepsilon_{t}$ can be associated with signal and noise respectively.

The distance between the $l$-dimensional subspace selected in step three of the basic SSA and the vectors $X_{j}$ in (1) should stay fairly small for $X_{j}, j>K$, if the time series $x_{t}, t=1, \ldots, T$ continutes for $t>T$ and there is no change in the mechanism generating $x_{t}$. Nonetheless, if at a certain time point $t+\tau$ the mechanism generating $x_{t}$ ($t>T+\tau$) has altered, then we can expect to see an increase in the distance between the $l$-dimensional subspace and the vectors $X_{j}$ for $j>K+\tau$. This is equivalent to saying that a change in the structure of the time series pushed the vectors $X_{j}$ out of the subspace.

Change point detection can be achieved by sequentially applying the SVD to the lag-covariance matrices computed in time intervals of length $N$, $[n+1, n+N]$, for each $n$ to accommodate the change point detection algorithm to slow changes in the time series structure.

Let $x_{1}, x_{2}, \ldots, x_{T}$ be a time series of length $T$. Let us choose two integers: the window width N (N≤T), and the lag parameter M (M≤N/2). Also, set K=N-M+1. The iterative change point detection algorithms has the following four steps.

**Step 1.** Construction of the *l*-dimensional space

1. For every suitable $n\geq0$ we construct the trajectory matrix considering the time interval [n+1, n+N]

$X_{B}^{(n)}= \left( \begin{matrix} x_{n+1} & x_{n+2} & x_{n+3} & \ldots& x_{n+K} \\ x_{n+2} & x_{n+3} & x_{n+4} & \ldots& x_{n+K+1} \\ \vdots& \vdots& \vdots& \ddots& \vdots\\ x_{n+M} & x_{n+M+1} & x_{n+M+2} & \ldots& x_{n+N} \end{matrix} \right)$ (3)

These matrices are called *base matrices*. The columns of the base matrix $X_{B}^{(n)}$ are vectors $X_{j}^{(n)}$:

$$X_{j}^{(n)}={(x_{n+j},\ldots,x_{n+j+M-1})}^{T}$$

2. For each n=0, 1,… we define the lag-covariance matrix $R_{n}=X_{B}^{(n)}{(X_{B}^{(n)})}^{T}$. The singular value decomposition of $R_{n}$ gives us a collection of M eigenvectors.

3. We select a distinct group $I=\{i_{1},\ldots,i_{l}\}$ of *l*<M of these eigenvectors; this determines an *l*-dimensional subspace $\mathcal{L}_{n,I}$ of the M-dimensional space $\mathbb{R}^{M}$ of the vectors$X_{j}^{(n)}$.

**Step 2.** Construction of the Test Matrix

Construct the matrix $X_{T}^{(n)}$ of size M$\times$Q, whose columns are vectors $X_{j}^{(n)}$, $(j=p+1,\ldots,p+Q)$; that is,

$X_{T}^{(n)}=\left( \begin{matrix} x_{n+p+1} & x_{n+p+2} & x_{n+p+3} & \ldots& x_{n+q} \\ x_{n+p+2} & x_{n+p+3} & x_{n+p+4} & \ldots& x_{n+q+1} \\ \vdots& \vdots& \vdots& \ddots& \vdots\\ x_{n+p+M} & x_{n+p+M+1} & x_{n+p+M+2} & \ldots& x_{n+q+M-1} \end{matrix} \right)$ (4)

where $q=p+Q$. This matrix is called *test matrix*.

**Step 3.** Computation of the Detection Statistics

The detection statistics are:

- $\mathcal{D}_{n,I,p,q}$, the sum of squared distances between the vectors $X_{j}^{(n)}$, $(j=p+1,\ldots,q)$ and the *l*-dimensional subspace $\mathcal{L}_{n,I}$ of $\mathbb{R}^{M}$ is calculated as following:

$\mathcal{D}_{n,I,p,q}=\sum_{j=p+1}^{q} {{(X}_{j}^{\left( n \right)})}^{T}X_{j}^{\left( n \right)}-{(X_{j}^{(n)})}^{T}UU^{T}X_{j}^{(n)}$ (5)

where $U$ is the $M\times l$ matrix whose columns $U_{i_{1}}, \ldots, U_{i_{l}}$ are the orthonormal eigenvectors that span the $\mathcal{L}_{n,I}$ subspace.

- $S_{n}={\tilde{\mathcal{D}}}_{n,I,p,q}/\mu_{n,I}$, the normalized sum of squares of distances. Here

${\tilde{\mathcal{D}}}_{n,I,p,q}=\frac{1}{MQ}\mathcal{D}_{n,I,p,q}$ (6)

and $\mu_{n,I}$ is an estimator of the normalized sum of squared distances ${\tilde{\mathcal{D}}}_{j,I,p,q}$ at the time intervals $\left[ j+1,j+m \right]$where the hypothesis of no change can be accepted. It is suggested to use $\mu_{n,I}={\tilde{\mathcal{D}}}_{m,I,0,K}$ where $m$ is the largest value of $m\leq n$ so that the hypothesis of no change has been accepted.

- Cumulative sum-type statistic

$W_{1}=S_{1}$, $W_{n+1}={max\{0,(W}_{n}+S_{n+1}-S_{n}-1/3MQ)\}$, $n\geq1$. (7)

**Step 4:** Decision Rule

The algorithm announces a structural change in the time series, if for some *n* we observe $W_{n}>h$ with the threshold $h=\frac{2t_{\alpha}}{MQ}\sqrt{\frac{1}{3}Q(3MQ-Q^{2}+1)}$, where $t_{\alpha}$ is the $(1-\alpha)$-quantile of the standard normal distribution.

**Choice of Parameters**: Window length N and lag M have to be chosen reasonably. The choice of N determines the smoothness or the effect of changes in the time series, i.e., if N is too large then we may miss changes in the time series. Alternatively, if N is too small we can have too many false alarms and outliers will be recognized as structural changes in the time Series. M is usually chosen to be M=N/2. The choice of $l$ is such that the largest $l$ principal components provide a good description of the signal and the lower $l-M$ components correspond to noise. It is advised to make a visual inspection of the SSA decomposition of the whole time series to choose $l$. A general recommendation for the choice of $p$ is that $p\geq K$ so that the columns of the base and test matrices do not coincide and thus, the change point detection algorithm is more sensitive to changes. In this study, we chose $N=20$, $M=10$, $p=K=11$, $q=12$ and $l=3$.

Fig. 9A shows the plot of the ordered set of eigenvalues of the lag-covariance matrix corresponding to the gene expression profile (time-series) of Cdkn2d. We can notice that the fourth and higher components only explain 5-6% of the cumulative variation (Fig 9B). Therefore, the first three largest eigenvalues of the lag-covariance matrix will provide a good description of the original time series for Cdkn2d. Hence, it is appropriate to group the largest three eigenvalues in set $I$ and the remaining eigenvalues in set $I^{'}$to decompose the time series of Cdkn2d into the main signal $z_{t}$ and noise $\varepsilon_{t}$.

Fig. 10 displays the decomposition of Cdkn2d time series into two separate time series that are reconstructed from the decomposition of the trajectory matrix $X$ into $X_{I}$ and $X_{I^{'}}$. $z_{t}$ is reconstructed from $X_{I}$ which corresponds to group $I$ of eigenvalues, and $X_{I^{'}}$ corresponds to group $I^{'}$ of the eigenvalues. It is obvious that time series $z_{t}$ that corresponds to the three largest principal components does a good job in describing the time series as it follows the main trend of the time series.

In the case of Cdkn2d time series, $l=3$ is chosen. Once $l$ is chosen the CPD algorithm is performed on the time series data. Fig. 11 displays the change points that are detected when CPD algorithm is performed on Cdkn2d time series. We can notice that the change point are declared when the major trend of the time series shifts.

Supplementary Text

Extended description of G1 phase results

**Myc:** In Fig. 4, we can see the connections Myc$\leftrightarrow$Cdc25a, Myc$\leftrightarrow$Cdkn2b, Myc$\leftarrow$Cdkn1b and Crebbp$\to$Myc. It is known that Cdc25a is capable of augmenting Myc-induced apoptosis in G1 [[5](#_ENREF_5)]. Myc represses cyclin dependent kinase inhibitors Cdkn2b during G1 arrest [[6](#_ENREF_6), [7](#_ENREF_7)] and takes part in Cdkn1b degradation [[8](#_ENREF_8), [9](#_ENREF_9)]. Crebbp is known to regulate and stabilize Myc in G1 to prevent inappropriate S phase entry [[10](#_ENREF_10), [11](#_ENREF_11)]. Furthermore, the Myc-Smad2 interaction is captured, while Myc is known to physically interact with Smad2 to inhibit TGF$\beta$ mediated induction of Cdkn2b in the G1 phase [[12](#_ENREF_12)].

**Smad2-4:** We observe the Smad2$\to$Cdkn1a, Smad4$\to$Cdkn1b, Smad2$\to$Rb1 and Skp2$\to$Smad2 connections in Fig. 4 (G1 phase). Studies show that Smad2 knockdown decreases Cdkn1a and releases G0/G1 arrest in mouse embryonic palate mesenchymal (MEPM) cells [[13](#_ENREF_13), [14](#_ENREF_14)]. Also, loss of Smad4 as a tumor suppressor is associated with Cdkn1b downregulation and decreases Rb1 phosphorylation that results in G1-S transition and cell proliferation [[15](#_ENREF_15)]. A recent study shows that Smad2 overexpression results in an increase in Rb1, leading to cell cycle arrest at the G1 to S phase boundary [[16](#_ENREF_16)]. Liu *et al.* (2007) have shown that Tgf$\beta$-induced Skp2 degredation is mediated by the Smad cascade, thereby facilitating cell cycle arrest at the G1/S transition [[17](#_ENREF_17)].

**Cyclin E/Cdk2:** The interaction of Ccne1 with Cdk2, Mcm3, Cdc45 and Cdc6 can be noted in Fig. 4. It is well-known that Ccne1 forms a complex with Cdk2, whose activity is required for the G1/S transition [[18](#_ENREF_18)]. Li *et al.* (2011) have indicated that Mcm3’s phosphorylation by Cyclin E is involved in its loading onto the chromatin during G1 phase and before DNA replication [[19](#_ENREF_19)] and that Cyclin E promotes chromatin loading of Cdc45 and phosphorylation of Cdc6 at the replication origins during the G1/S transition [[20](#_ENREF_20), [21](#_ENREF_21)]. We can also notice the Cdk2$\to$Trp53 interaction where it’s been shown the activation of Trp53 tumor suppressor is required for Cdk2 phosphorylation and progression through G1 phase [[22](#_ENREF_22), [23](#_ENREF_23)].

**Pcna:** The interaction of Pcna with Gadd45a and Trp53 can be observed in Fig. 4. Multiple studies have shown that Gadd45a binds to and interacts with Pcna [[24-26](#_ENREF_24)] and inhibits entry of cell into S phase [[27](#_ENREF_27)]. Furthermore, studies have shown that Trp53 mediates the activation of Pcna expression leading to arrest of cell growth at late G1 phase [[28-30](#_ENREF_28)].

**Abl1 and Hdac2:** We can note the Abl1$\leftrightarrow$Mdm2, Bub3$\to$ Hdac2 in Fig. 4. Research has revealed the role of Abl1 in phosphorylation of Mdm2 which neutralizes the inhibitory effect of Mdm2 on Trp53 in response to DNA damage and stabilizes p53 in an active form [[31-33](#_ENREF_31)]. Yoon *et al.* (2004) have indicated that Bub3 directly interact with Hdac2 sauggesting that the Bub3–HDAC complexes are constituitively present thoughout G1 and G2 phases and may interact with Mad1l1 [[34](#_ENREF_34)].

Extended description of S phase results

**Pre-replicative complex**: We can see the interaction of Mcm3 with Cdc45 in Fig. 5. Mcm3 and Cdc45, both of which are interacting components of the pre-replicative complex [[35-37](#_ENREF_35)] are known to dissociate from the origin DNA and associate with non-origin DNA and move with replication forks at the beginning of S phase [[38](#_ENREF_38), [39](#_ENREF_39)]. In addition, Cdc45’s loading onto the chromatin in the S phase is required to activate the helicase activity of the MCM complex [[40](#_ENREF_40), [41](#_ENREF_41)]. Cdc6 has been shown to activate Cdk2 to initiate DNA replication and G1-S phase progression [[21](#_ENREF_21), [42](#_ENREF_42)], captured by the edge Cdc6$\to$Cdk2 in Fig. 5. Cdc6 is also known to activate Cdk2 to prevent re-replication during S and G2 phases [[43](#_ENREF_43)]. Dbf4$\to$Cdk1 can be seen in Fig. 5; Cdk1 is known to target the Dbf4-Cdc7 kinase at the end of S phase to prevent re-replication in G2/M [[44](#_ENREF_44), [45](#_ENREF_45)].

**Mdm2:** The Cdk1$\to$Mdm2 and Ttk$\to$Mdm2 can be detected in Fig. 5. Mdm2 is known to be phosphorylated by Cyclin A-Cdk1 complexes at the onset of S phase to reduce its interaction with Trp53 [[46](#_ENREF_46)]. Moreover, Ttk phosphorylates Mdm2 which facilitates oxidative DNA damage repair and cell survival during S-phase [[47](#_ENREF_47)].

Extended description of G2/M phase results

**Cdc25b:** The Cdc25b$\to$Plk1 and Cdk1$\to$Cdc25b edges can be noticed in Fig. 6. Studies have concluded that Plk1 not only plays a critical role in mitotic entry and progression through mitosis, but also regulates mitotic exit by phosphorylating Cdc25b which is an activator of APC/C [[48](#_ENREF_48), [49](#_ENREF_49)]. Cdc25 phosphatases are known to be tightly coupled with dephosphorylation and complex assembly of Cdk1 which plays an essential role in the activation of Cdk1 at G2/M [[50](#_ENREF_50), [51](#_ENREF_51)]. On the other hand, an important component of cell cycle regulation at G2/M transition is that Cdk1 phosphorylation targets Cdc25b for degradation [[52](#_ENREF_52)].

**Cdh1:** We can notice the Cdh1$\to$Dbf4 in Fig. 6. Yamada *et al.* (2013) have shown that Dbf4 is targeted for degardation by Cdh1 between late M and early G1 phase which is when DBf4 levels decline during the cell cycle [[53](#_ENREF_53)].

**Plk1:** The Plk1$\to$Cdc20 and Plk1$\to$Pttg1 edges can be noticed in Fig. 6. Plk1 phosphorylates Cdc20 as an essential step in the activation of anaphase promoting complex (APC/C) leading to the release of Espl1 to trigger the cleavage of cohesin and separation of sister chromatids [[54](#_ENREF_54), [55](#_ENREF_55)]. Furthermore, reports show that Pttg1 modulates the mitotic role of the Polo-like kinase (Plk) pathway [[56](#_ENREF_56)].

Supplementary Table S1. List of time-dependent biological processes according to the Reactome pathway database.

|  | **Biological Process** | **Members (Genes)** |
| --- | --- | --- |
| **1** | G0 and Early G1 | Cdk2; E2f4; Rbl1; Tfdp1; Ccne1 |
| **2** | G1 Phase | Cdkn2b; Cdkn2c; E2f4; Skp2; E2f1; Cdkn2d; Ccnd1; Cdkn1b; Cdkn1a; Rb1; Cdkn2a; Cdk4; Ccnh; Tfdp1; Rbl1 |
| **3** | p53-Dependent G1 DNA Damage Response | Ccne1; Trp53; Cdkn1b; Cdkn1a; Atm; Cdk2; Mdm2 |
| **4** | G1/S Transition | Rb1; Skp2; E2f1; Cdkn1b; Cdc25a; Ccne1; Pkmyt1; Orc1; Cdk1; Cdk2; Dbf4; Tfdp1; Cdc45; Mcm3; Wee1; Ccnh; Cdkn1a; Cdc6; Cdc7 |
| **5** | G1/S DNA Damage Checkpoints | Ccne1; Trp53; Cdkn1b; Cdc25a; Atm; Cdk2; Mdm2; Cdkn1a; Chek1 |
| **6** | p53-Independent DNA Damage Response | Cdc25a; Atm; Chek1 |
| **7** | DNA Repair | Pcna; Atm; Ccnh; Prkdc |
| **8** | DNA Replication | Pcna; Cdkn1b; Cdkn1a; Rb1; Orc1; Cdk2; Dbf4; Cdc45; Mcm3; Cdc6; Cdc7 |
| **9** | Unwinding of DNA | Cdc45; Mcm3 |

Supplementary Table S1 (Contd.). List of time-dependent biological processes according to the Reactome pathway database.

|  | **Biological Process** | **Members (Genes)** |
| --- | --- | --- |
| **10** | Removal of licensing factors from origins | Cdkn1b; Cdkn1a; Rb1; Orc1; Cdk2; Mcm3; Cdc6 |
| **11** | G2/M DNA replication checkpoint | Cdk1; Wee1; Pkmyt1; Ccnb2 |
| **12** | Mitotic G2-G2/M phases | Ccnb2; E2f1; Plk1; Cdc25b; Cdc25a; Pkmyt1; Cdk1; Cdk2; Wee1; Ccnh |
| **13** | G2/M Transition | Ccnb2; Plk1; Cdc25b; Cdc25a; Pkmyt1; Cdk1; Cdk2; Wee1; Ccnh |
| **14** | Mitotic Spindle Checkpoint | Mad2l1; Bub3; Bub1b; Mad1l1; Cdc20; Anapc1 |
| **15** | Separation of Sister Chromatids | Smc1a; Mad2l1; Bub1; Bub3; Bub1b; Mad1l1; Smc3; Stag1; Cdc20; Plk1; Espl1; Anapc1; Pttg1; Rad21 |
| **16** | M/G1 Transition | Orc1; Cdk2; Dbf4; Cdc45; Mcm3; Cdc6; Cdc7 |

Supplementary Table S2. Statistics for the reconstructed network of the G1, S and G2/M phases incorporating transcription factors in the analysis.

| Reconstructed Network | Precision | False Discovery Rate |
| --- | --- | --- |
| G1 phase | 0.72 | 0.28 |
| S phase | 0.67 | 0.33 |
| G2/M phase | 0.66 | 0.34 |

**Supplementary References**

1. Cooper S: Mammalian cells are not synchronized in G1‐phase by starvation or inhibition: considerations of the fundamental concept of G1‐phase synchronization. *Cell proliferation* 1998, 31:9-16.

2. Cooper S: Rethinking synchronization of mammalian cells for cell cycle analysis. *Cellular and Molecular Life Sciences CMLS* 2003, 60:1099-1106.

3. Boker SM, Rotondo JL, Xu M, King K: Windowed cross-correlation and peak picking for the analysis of variability in the association between behavioral time series. *Psychological Methods* 2002, 7:338.

4. Moskvina V, Zhigljavsky A: Application of the singular spectrum analysis for change-point detection in time series. *Journal of Time Series Analysis, submitted* 2001.

5. Macdonald K, Bennett MR: cdc25A is necessary but not sufficient for optimal c-myc–induced apoptosis and cell proliferation of vascular smooth muscle cells. *Circulation research* 1999, 84:820-830.

6. Seoane J, Pouponnot C, Staller P, Schader M, Eilers M, Massagué J: TGFβ influences Myc, Miz-1 and Smad to control the CDK inhibitor p15INK4b. *Nature cell biology* 2001, 3:400-408.

7. Staller P, Peukert K, Kiermaier A, Seoane J, Lukas J, Karsunky H, Möröy T, Bartek J, Massagué J, Hänel F: Repression of p15INK4b expression by Myc through association with Miz-1. *Nature cell biology* 2001, 3:392-399.

8. Lutz W, Leon J, Eilers M: Contributions of Myc to tumorigenesis. *Biochimica Et Biophysica Acta (BBA)-Reviews on Cancer* 2002, 1602:61-71.

9. Sicari BM, Troxell R, Salim F, Tanwir M, Takane KK, Fiaschi-Taesch N: c-myc and skp2 coordinate p27 degradation, vascular smooth muscle proliferation, and neointima formation induced by the parathyroid hormone-related protein. *Endocrinology* 2011, 153:861-872.

10. Faiola F, Liu X, Lo S, Pan S, Zhang K, Lymar E, Farina A, Martinez E: Dual regulation of c-Myc by p300 via acetylation-dependent control of Myc protein turnover and coactivation of Myc-induced transcription. *Molecular and cellular biology* 2005, 25:10220-10234.

11. Rajabi HN, Baluchamy S, Kolli S, Nag A, Srinivas R, Raychaudhuri P, Thimmapaya B: Effects of depletion of CREB-binding protein on c-Myc regulation and cell cycle G1-S transition. *Journal of Biological Chemistry* 2005, 280:361-374.

12. Feng X-H, Liang Y-Y, Liang M, Zhai W, Lin X: Direct interaction of c-Myc with Smad2 and Smad3 to inhibit TGF-β-mediated induction of the CDK inhibitor p15 Ink4B. *Molecular cell* 2002, 9:133-143.

13. Koinuma D, Tsutsumi S, Kamimura N, Taniguchi H, Miyazawa K, Sunamura M, Imamura T, Miyazono K, Aburatani H: Chromatin immunoprecipitation on microarray analysis of Smad2/3 binding sites reveals roles of ETS1 and TFAP2A in transforming growth factor β signaling. *Molecular and cellular biology* 2009, 29:172-186.

14. Wang M, Huang H, Chen Y: Smad2/3 is involved in growth inhibition of mouse embryonic palate mesenchymal cells induced by all‐trans retinoic acid. *Birth Defects Research Part A: Clinical and Molecular Teratology* 2009, 85:780-790.

15. Zhang B, Chen X, Bae S, Singh K, Washington M, Datta P: Loss of Smad4 in colorectal cancer induces resistance to 5-fluorouracil through activating Akt pathway. *British journal of cancer* 2014, 110:946-957.

16. Alotaibi M, Kitase Y, Shuler C: smad2 Overexpression reduces the proliferation of the Junctional Epithelium. *Journal of dental research* 2014:0022034514543016.

17. Liu W, Wu G, Li W, Lobur D, Wan Y: Cdh1-anaphase-promoting complex targets Skp2 for destruction in transforming growth factor β-induced growth inhibition. *Molecular and cellular biology* 2007, 27:2967-2979.

18. Koff A, Giordano A, Desai D, Yamashita K, Harper JW, Elledge S, Nishimoto T, Morgan DO, Franza BR, Roberts JM: Formation and activation of a cyclin E-cdk2 complex during the G1 phase of the human cell cycle. *Science* 1992, 257:1689-1694.

19. Li J, Deng M, Wei Q, Liu T, Tong X, Ye X: Phosphorylation of MCM3 protein by cyclin E/cyclin-dependent kinase 2 (Cdk2) regulates its function in cell cycle. *Journal of Biological Chemistry* 2011, 286:39776-39785.

20. Ferguson RL, Maller JL: Centrosomal localization of cyclin E-Cdk2 is required for initiation of DNA synthesis. *Current Biology* 2010, 20:856-860.

21. Jiang W, Wells NJ, Hunter T: Multistep regulation of DNA replication by Cdk phosphorylation of HsCdc6. *Proceedings of the National Academy of Sciences* 1999, 96:6193-6198.

22. Zalzali H, Nasr B, Harajly M, Basma H, Ghamloush F, Ghayad S, Ghanem N, Evan GI, Saab R: CDK2 Transcriptional Repression Is an Essential Effector in p53-Dependent Cellular Senescence—Implications for Therapeutic Intervention. *Molecular Cancer Research* 2015, 13:29-40.

23. Nevis KR, Cordeiro-Stone M, Cook JG: Origin licensing and p53 status regulate Cdk2 activity during G1. *Cell Cycle* 2009, 8:1952-1963.

24. Vairapandi M, Azam N, Balliet AG, Hoffman B, Liebermann DA: Characterization of MyD118, Gadd45, and proliferating cell nuclear antigen (PCNA) interacting domains PCNA impedes MyD118 and Gadd45-mediated negative growth control. *Journal of Biological Chemistry* 2000, 275:16810-16819.

25. Hall PA, Kearsey JM, Coates PJ, Norman DG, Warbrick E, Cox LS: Characterisation of the interaction between PCNA and Gadd45. *Oncogene* 1995, 10:2427-2433.

26. Chen I-T, Smith ML, O'Connor PM, Fornace Jr AJ: Direct interaction of Gadd45 with PCNA and evidence for competitive interaction of Gadd45 and p21Waf1/Cip1 with PCNA. *Oncogene* 1995, 11:1931-1937.

27. Smith ML, Chen I-T, Zhan Q, Bae I, Chen C-Y, Gilmer TM, Kastan MB, O'Connor PM, Fornace AJ: Interaction of the p53-regulated protein Gadd45 with proliferating cell nuclear antigen. *Science* 1994, 266:1376-1380.

28. Morris GF, Bischoff JR, Mathews MB: Transcriptional activation of the human proliferating-cell nuclear antigen promoter by p53. *Proceedings of the National Academy of Sciences* 1996, 93:895-899.

29. Shivakumar CV, Brown DR, Deb S, Deb SP: Wild-type human p53 transactivates the human proliferating cell nuclear antigen promoter. *Molecular and Cellular Biology* 1995, 15:6785-6793.

30. Xu J, Morris GF: p53-mediated regulation of proliferating cell nuclear antigen expression in cells exposed to ionizing radiation. *Molecular and cellular biology* 1999, 19:12-20.

31. Goldberg Z, Sionov RV, Berger M, Zwang Y, Perets R, Van Etten RA, Oren M, Taya Y, Haupt Y: Tyrosine phosphorylation of Mdm2 by c‐Abl: implications for p53 regulation. *The EMBO journal* 2002, 21:3715-3727.

32. Sionov RV, Moallem E, Berger M, Kazaz A, Gerlitz O, Ben-Neriah Y, Oren M, Haupt Y: c-Abl neutralizes the inhibitory effect of Mdm2 on p53. *Journal of Biological Chemistry* 1999, 274:8371-8374.

33. Zuckerman V, Lenos K, Popowicz GM, Silberman I, Grossman T, Marine J-C, Holak TA, Jochemsen AG, Haupt Y: c-Abl phosphorylates Hdmx and regulates its interaction with p53. *Journal of Biological Chemistry* 2009, 284:4031-4039.

34. Yoon Y-M, Baek K-H, Jeong S-J, Shin H-J, Ha G-H, Jeon A-H, Hwang S-G, Chun J-S, Lee C-W: WD repeat‐containing mitotic checkpoint proteins act as transcriptional repressors during interphase. *FEBS letters* 2004, 575:23-29.

35. Maric M, Maculins T, De Piccoli G, Labib K: Cdc48 and a ubiquitin ligase drive disassembly of the CMG helicase at the end of DNA replication. *Science* 2014, 346:1253596.

36. Bruck I, Kaplan DL: GINS and Sld3 compete with one another for Mcm2-7 and Cdc45 binding. *Journal of Biological Chemistry* 2011, 286:14157-14167.

37. Hardy CF: Identification of Cdc45p, an essential factor required for DNA replication. *Gene* 1997, 187:239-246.

38. Aparicio OM, Weinstein DM, Bell SP: Components and dynamics of DNA replication complexes in S. cerevisiae: redistribution of MCM proteins and Cdc45p during S phase. *Cell* 1997, 91:59-69.

39. Tanaka T, Knapp D, Nasmyth K: Loading of an Mcm protein onto DNA replication origins is regulated by Cdc6p and CDKs. *Cell* 1997, 90:649-660.

40. Zou L, Stillman B: Formation of a preinitiation complex by S-phase cyclin CDK-dependent loading of Cdc45p onto chromatin. *Science* 1998, 280:593-596.

41. Masuda T, Mimura S, Takisawa H: CDK‐and Cdc45‐dependent priming of the MCM complex on chromatin during S‐phase in Xenopus egg extracts: possible activation of MCM helicase by association with Cdc45. *Genes to Cells* 2003, 8:145-161.

42. Lunn CL, Chrivia JC, Baldassare JJ: Activation of Cdk2/Cyclin E complexes is dependent on the origin of replication licensing factor Cdc6 in mammalian cells. *Cell Cycle* 2010, 9:4533-4541.

43. Petersen BO, Lukas J, Sørensen CS, Bartek J, Helin K: Phosphorylation of mammalian CDC6 by cyclin A/CDK2 regulates its subcellular localization. *The EMBO Journal* 1999, 18:396-410.

44. Knockleby J, Kim BJ, Lee H: Cdk1 prevents DNA rereplication in G2/M by phosphorylating and facilitating the removal of Cdc7 from chromatin at the end of S phase. *Cancer Research* 2013, 73:575-575.

45. Nougarède R, Della Seta F, Zarzov P, Schwob E: Hierarchy of S-phase-promoting factors: yeast Dbf4-Cdc7 kinase requires prior S-phase cyclin-dependent kinase activation. *Molecular and Cellular Biology* 2000, 20:3795-3806.

46. Hu W, Feng Z, Levine AJ: The regulation of multiple p53 stress responses is mediated through MDM2. *Genes & cancer* 2012, 3:199-208.

47. Yu Z-C, Huang Y-F, Shieh S-Y: Requirement for human Mps1/TTK in oxidative DNA damage repair and cell survival through MDM2 phosphorylation. *Nucleic acids research* 2015:gkv1173.

48. Lobjois V, Jullien D, Bouché J-P, Ducommun B: The polo-like kinase 1 regulates CDC25B-dependent mitosis entry. *Biochimica et Biophysica Acta (BBA)-Molecular Cell Research* 2009, 1793:462-468.

49. Lobjois V, Froment C, Braud E, Grimal F, Burlet-Schiltz O, Ducommun B, Bouche J-P: Study of the docking-dependent PLK1 phosphorylation of the CDC25B phosphatase. *Biochemical and biophysical research communications* 2011, 410:87-90.

50. Timofeev O, Cizmecioglu O, Settele F, Kempf T, Hoffmann I: Cdc25 phosphatases are required for timely assembly of CDK1-cyclin B at the G2/M transition. *Journal of Biological Chemistry* 2010, 285:16978-16990.

51. Kumagai A, Dunphy WG: The cdc25 protein controls tyrosine dephosphorylation of the cdc2 protein in a cell-free system. *Cell* 1991, 64:903-914.

52. Baldin V, Cans C, Knibiehler M, Ducommun B: Phosphorylation of human CDC25B phosphatase by CDK1-cyclin A triggers its proteasome-dependent degradation. *Journal of Biological Chemistry* 1997, 272:32731-32734.

53. Yamada M, Watanabe K, Mistrik M, Vesela E, Protivankova I, Mailand N, Lee M, Masai H, Lukas J, Bartek J: ATR–Chk1–APC/CCdh1-dependent stabilization of Cdc7–ASK (Dbf4) kinase is required for DNA lesion bypass under replication stress. *Genes & development* 2013, 27:2459-2472.

54. Eckerdt F, Strebhardt K: Polo-Like Kinase 1: Target and Regulator of Anaphase-Promoting Complex/Cyclosome–Dependent Proteolysis. *Cancer research* 2006, 66:6895-6898.

55. Hyun S-Y, Sarantuya B, Lee H-J, Jang Y-J: APC/C Cdh1-dependent degradation of Cdc20 requires a phosphorylation on CRY-box by Polo-like kinase-1 during somatic cell cycle. *Biochemical and biophysical research communications* 2013, 436:12-18.

56. Bouchet A, Sakakini N, Atifi ME, Clec'h C, Bräuer‐Krisch E, Rogalev L, Laissue JA, Rihet P, Le Duc G, Pelletier L: Identification of AREG and PLK1 pathway modulation as a potential key of the response of intracranial 9L tumor to microbeam radiation therapy. *International Journal of Cancer* 2015, 136:2705-2716.
